# EpiAge: a next-generation sequencing-based *ELOVL2* epigenetic clock for biological age assessment in saliva and blood across health and disease

**DOI:** 10.18632/aging.206188

**Published:** 2025-01-22

**Authors:** David Cheishvili, Sonia Do Carmo, Filippo Caraci, Margherita Grasso, A Claudio Cuello, Moshe Szyf

**Affiliations:** 1 EpiMedTech Global, Singapore 409051, Singapore; 2 HKG Epitherapeutics Ltd., Hong Kong SAR, China; 3 Gerald Bronfman Department of Oncology, McGill University, Montreal H4A 3T2, Canada; 4 Department of Pharmacology & Therapeutics, McGill University, Montreal H3G 1Y6, Canada; 5 Visiting Professor, Department of Pharmacology, Oxford University, Oxford OX13QT, UK; 6 Department of Drug and Health Sciences, University of Catania, Catania 95125, Italy; 7 Neuropharmacology and Translational Neurosciences Research Unit, Oasi Research Institute-IRCCS, Troina 94018, Italy

**Keywords:** epigenetic clock, elovl2, next-generation sequencing, EpiAge, Alzheimer's disease

## Abstract

This study introduces EpiAgePublic, a new method to estimate biological age using only three specific sites on the gene *ELOVL2,* known for its connection to aging. Unlike traditional methods that require complex and extensive data, our model uses a simpler approach that is well-suited for next-generation sequencing technology, which is a more advanced method of analyzing DNA methylation. This new model overcomes some of the common challenges found in older methods, such as errors due to sample quality and processing variations.

We tested EpiAgePublic with a large and varied group of over 4,600 people to ensure its accuracy. It performed on par with, and sometimes better than, more complicated models that use much more data for age estimation. We examined its effectiveness in understanding how factors like HIV infection and stress affect aging, confirming its usefulness in real-world clinical settings.

Our results prove that our simple yet effective model, EpiAgePublic, can capture the subtle signs of aging with high accuracy. We also used this model in a study involving patients with Alzheimer’s Disease, demonstrating the practical benefits of next-generation sequencing in making precise age-related assessments.

This study lays the groundwork for future research on aging mechanisms and assessing how different interventions might impact the aging process using this clock.

## INTRODUCTION

The broad interest in *ELOVL2* (Elongation of Very Long Chain Fatty Acids Protein 2) in the field of aging research is highlighted by more than a hundred scholarly articles discussing its link with epigenetic aging. This extensive body of research confirms its critical role and widespread recognition within the scientific community. Consistently heralded as a potent epigenetic biomarker, *ELOVL2* has proven indispensable in elucidating the biological processes associated with aging [1, 2]. *ELOVL2* has been integrated into epigenetic clocks to accurately determine age-related differences across various disease groups, highlighting its utility in age prediction [3]. Research indicates that *ELOVL2* affects the aging process through its role in regulating lipid metabolism, with its epigenetic alterations closely linked to age prediction capabilities (Li et al., 2022). Additionally, the CpG sites within *ELOVL2’s* regulatory regions play a crucial role in age prediction models, underscoring the gene’s central importance in epigenetic clocks [4]. Since it is well known that there is a close correlation in methylation between juxtaposed CpG positions [5–8], we reasoned that including additional CpGs in the region could increase the accuracy of the analysis. Therefore, we designed the primers to cover not only the three CpGs discussed (cg16867657, cg21572722, and cg24724428) but also to capture ten additional CpGs within the same region.

Further research has shown that DNA methylation patterns of *ELOVL2* are associated with age-related macular degeneration, establishing it as a reliable biomarker for aging in ocular tissues (Saptarshi et al., 2021). In addition, *ELOVL2* methylation has been utilized in forensic science for age estimation, which underscores its critical value in age prediction applications (Brenna & Kothapalli, 2021).

In summary, *ELOVL2* is a fundamental gene in the development of epigenetic clocks and age prediction models. Its methylation patterns serve as a robust indicator of biological age, demonstrating significant utility across various research and clinical contexts.

Nevertheless, traditional epigenetic clocks primarily utilize Illumina Infinium BeadChips, which analyze hundreds of thousands of CpG sites. However, this approach is prone to technical variances from sample preparation, probe hybridization, chemistry, and batch effects, which often compromise data reliability [9]. Despite efforts to mitigate these issues through normalization and batch correction, significant challenges persist, particularly affecting the clocks' utility in both basic and translational research, including short-term longitudinal studies like clinical trials.

Next-generation sequencing (NGS) significantly enhances the precision and scope of epigenetic studies by addressing the limitations of traditional array technologies, such as technical noise and reliance on predefined sequences. With its high-throughput capability, base-resolution accuracy, and broader genomic coverage, NGS not only allows for detailed examination of methylation patterns across diverse genomic contexts but also facilitates the discovery of novel methylation sites. This comprehensive approach allows for more accurate identification of differentially methylated regions, providing a robust alternative that surpasses traditional methods in both detail and scalability [10]. The accuracy of DNA methylation determination of bisulfite-converted DNA by next-generation sequencing is dependent on the number of reads to achieve statistical power because of the heterogeneity of methylation profiles even in the same tissue. Therefore, reducing the number of regions that are required to be sequenced for a given biomarker would increase the depth of sequencing and, thus, the power for accurate determination of its DNA methylation, especially when samples are multiplexed, to increase the cost-effectiveness of the biomarker. We, therefore, examined in this study whether we need the hundreds or thousands of regions that are used in all current epigenetic clocks to develop an accurate and cost-effective epigenetic clock.

Most current clocks use blood as a biological sample. Saliva has become a valuable resource for epigenetic age estimation due to its non-invasive collection, ease of handling, and rich DNA content from both epithelial and white blood cells. This mixed cellular composition captures systemic biological signals, reflecting both oral health and broader immune responses, making it suitable for large-scale studies and clinical settings where less invasive methods enhance participant compliance. Additionally, saliva mirrors the methylome of blood and other tissues, which supports its use in epigenetic studies exploring the effects of environmental exposures, lifestyle, and disease states on aging [11]. Research shows saliva's relevance in studying various health conditions, such as its association with Parkinson's disease and certain carcinomas through differential methylation patterns [12, 13]. Additionally, saliva has been utilized to evaluate epigenetic age acceleration, with adjustments made for cell type proportions in the samples [14]. It has also been instrumental in developmental studies linking birth weight with DNA methylation [15] and evaluating childhood BMI and social disparities through epigenetic markers [16].

This utility highlights saliva's potential to advance our understanding of biological aging and age-related diseases. In this study, we assess the performance of various biological clocks in saliva versus blood.

This study introduces EpiAgePublic, a new epigenetic aging model that leverages just three strategically selected CpG sites within the *ELOVL2* gene, a key marker strongly associated with aging. Inspired by minimal-marker models like the “Epigenetic age-predictor for mice based on three CpG sites [17], EpiAgePublic is optimized for next-generation sequencing technologies. Unlike traditional array-based clocks that rely on hundreds to thousands of CpG sites, this model demonstrates the effectiveness of using fewer markers to accurately predict age, thus addressing common limitations of older methods. This method not only mitigates issues related to DNA quality and batch effects but also enhances the model’s specificity and clinical applicability.

Validated against a comprehensive dataset comprising 4,625 individuals, EpiAgePublic has shown predictive accuracies on par with, or even exceeding, those of well-established epigenetic clocks such as DNAmAge (Horvath’s Clock) and DNAmPhenoAge. The model’s performance was rigorously evaluated in various clinical contexts, including its correlation with HIV infection and stress levels, underscoring its utility alongside more traditional, complex clocks.

Our results affirm that EpiAgePublic, despite its simplicity in focusing on only three CpG sites, effectively captures the biological intricacies of aging. This demonstrates its viability as a powerful tool for aging research and clinical application. We also determined that the clock performs well on saliva samples. We next developed a targeted amplification-next-generation sequencing assay that captures 13 CpG sites around the *ELOVL2* gene and used a proprietary model on DNA methylation data from the *ELOVL2* region. The model was further employed in a clinical study on Alzheimer’s Disease, where next-generation sequencing was utilized to assess epigenetic age. This application not only highlighted EpiAgePublic’s precision but also illustrated the significant advantages of next-generation sequencing in enhancing the accuracy of epigenetic age evaluations. By using saliva as the biological sample, we increase the feasibility of wide usage of this clock.

## MATERIALS AND METHODS

### Estimation of DNA methylation age

The Horvath’s DNAmAge, Hannum’s DNAmAgeHannum, DNAmPhenoAge, DNAmAgeSkinBloodClock, and DNA GrimAge (both versions 1 and 2) methylation ages were determined using the online DNA Methylation Age Calculator, provided by the Clock Foundation: https://dnamage. clockfoundation.org/.

EpiAge for next-generation sequencing data from individuals in the Alzheimer’s Disease study was obtained through a commercial service provided by HKG epiTherapeutics. This service utilizes a proprietary model based on DNA methylation data within the same *ELOVL2* region discussed in this paper, specifically designed for next-generation sequencing applications. The analysis was performed in triplicate.

### Development and application of the EpiAgePublic model to investigate biological aging dynamics

In this study, we developed the EpiAgePublic model to explore the impact of various clinical outcomes on biological aging. Linear regression was employed to assign weights to three targeted CpG sites: cg16867657, cg21572722, and cg24724428. We selected these three CpG sites from a set of 13 CpGs used in our previous study [18] due to their presence on standard DNA methylation microarray platforms. This enabled us to validate the model across a wide range of publicly available datasets derived from these platforms. The model calculates beta values using the formula:


$$\begin{array}{c} (\beta_{cg16867657}\;x\;122.70+\beta_{cg21572722}\;x\;\\ 24.45+\beta_{cg24724428}\times (-30.44))-42.91. \end{array}$$

### Model training

The model was trained using a comprehensive DNA methylation dataset aggregated from four public databases: GSE55763, GSE157131, GSE40279, and GSE30870, as detailed in Table 1. This training dataset, derived from Illumina 450K and Epic array platforms, encompasses a demographic range of individuals aged 0 to 103 years, including Caucasian-European, Hispanic Mexican, and African American ethnic groups. The sex distribution was balanced, comprising 2506 males, 2079 females, and 40 individuals with unspecified gender.

**Table 1:** Comprehensive dataset summary for epigenetic age model development and clinical studies.

**GSE ID**	**Sample tissue type**	**Total samples**	**Age range**	**Cohort description**	**Methylation platform**	**Dataset application**	**Purpose**
GSE55763	Blood	2711	23.7 - 75	General Population	450K	epiAgePublic model	Training
GSE157131	Blood	1218	26.41 - 94.74	General Population	450K/Epic	epiAgePublic model	Training
GSE40279	Blood	656	31 - 68	General Population	450K	epiAgePublic model	Training
GSE30870	Blood	40	30 - 48	General Population	450K	epiAgePublic model	Training
GSE78874	Saliva	258	36-88	General Population	450K	epiAgePublic validation	Validation
GSE150643	Saliva	240	9.19-15.85	General Population	450K	epiAgePublic validation	Validation
GSE92767	Saliva	54	18-73	General Population	450K	epiAgePublic validation	Validation
GSE99029	Saliva	57	21-91	General Population	450K	epiAgePublic validation	Validation
GSE67751	Blood	92	24 - 68	Healthy and HIV Patients	450K	HIV	Validation
GSE117859	Blood	608	25 - 75	HIV Patients	450K	HIV	Validation
GSE53840	Blood	120	31 - 68	HIV Patients	450K	HIV	Validation
GSE185391	Blood	86	30 - 48	HIV Patients	450K	HIV treatment	Validation
GSE167202	Blood	525	17-96	Healthy, COVID-19 and other infections	Epic	COVID-19	Validation
GSE168739	Blood	407	19-61	COVID-19 patients	Epic	COVID-19	Validation
GSE72680	Blood	422	18-77	Trauma and Psychiatric Symptoms in African Americans	450K	Stress	Validation
GSE128235	Blood	533	18-87	Depression	450K	Controls, Depression	Validation
GSE125105	Blood	847	17-87	Depression	450K	Controls, Depression	Validation
GSE144858	Blood	300	52-90	Alzheimer’s	450K	Controls, MCI, Alzheimer’s	Validation

The selection of this diverse dataset was critical for developing an inclusive and representative EpiAgePublic model. The model achieved an R-squared value of 0.7512 in the training cohort, indicating its robust ability to accurately reflect the relationship between DNA methylation patterns and biological age.

### Model validation

After development, the EpiAgePublic model was validated on independent datasets, which included several additional public cohorts listed in Table 1. These validation datasets were not part of the training cohort and were used to test the generalizability and robustness of the model across different populations and clinical contexts. The validation results showed that the model maintained high accuracy and robustness, supporting its effectiveness as a reliable indicator of biological aging across diverse populations.

### Epigenetic age acceleration (EAA)

Epigenetic Age Acceleration (EAA) measures biological aging dynamics. It is computed as the difference between DNA methylation age and chronological age. A positive EAA value indicates that an individual’s biological age surpasses their chronological age, suggesting accelerated aging. This metric is used to compare aging rates across different clinical conditions by analyzing the differences in EAA between a specific condition and a control group.

### Data source

The DNA methylation data used in this study were derived from publicly available datasets and newly collected original data. The Down syndrome and Alzheimer’s Disease cohorts represent the original data collected specifically for this study. Details regarding the sample collection, processing, and DNA extraction for these cohorts are provided below.

### Demographic characteristics of the study sample cohorts Down syndrome study

The study protocol was approved by the Institutional Review Board (IRB) of the Oasi Research Institute—IRCCS, Troina, Italy (Protocol Number: 2016/1.0/122/CE-IRCCS-OASI). Participants or their legal representatives gave written informed consent, as approved by the Ethics Committee of the Oasi Research Institute-IRCCS. This study included 22 control participants (9 males and 13 females) and 22 individuals with Down syndrome (9 males and 13 females). The mean age for the control group was 40.95 years (SD = 9.276), and for the Down syndrome group, it was 40.65 years (SD = 8.969). An unpaired parametric t-test showed no significant age difference between the two groups (p-value = 0.9146).

### Alzheimer’s disease study

This research was approved by the IRB of Oasi Research Institute—IRCCS, Troina, Italy, under Protocol Number: 2019/03/18/CE-IRCCS-OASI/18. Participants or their legal representatives gave written informed consent, as approved by the Ethics Committee of the Oasi Research Institute-IRCCSS. The study involved 55 participants, with a demographic composition of 10 males and 17 females in the control group and 7 males and 21 females in the Alzheimer’s disease (AD) group. The mean age was 72.25 years (SD = 9.26) for the controls and 73.01 years (SD = 8.91) for the AD group. Due to technical difficulties, methylation analysis was performed on 54 participants, excluding one from the original cohort. An unpaired parametric t-test revealed that the age difference between the two groups was not statistically significant (p-value = 0.0585), although this value approaches the conventional threshold for significance.

### Plasma sample processing and **white blood cell isolation**

Plasma samples were collected according to standard procedures. Briefly, fasting venous blood was collected in lavender EDTA-K2 BD vacutainer tubes and centrifuged at 1900 rpm for 10 minutes for the separation of the plasma component from the cellular constituents. After a second centrifugation (3900 rpm for 10 min) to purify from biological debris, plasma samples were separated in aliquots and stored at –80° C until use. The blood was used for peripheral blood mononuclear cells (PBMCs) isolation by using the Lympholyte®-H density gradient separation medium (Cedarlane, Burlington, NC, USA) according to the manufacturer’s instructions, with slight modifications. Briefly, the blood was diluted with an equal volume of 1X PBS, mixed gently and added to one part of Lympholyte®-H. After a centrifugation step (400 rcf for 30 min), a well-defined lymphocyte layer appeared at the interface, which was removed and transferred by using a serological pipette to a new sterile centrifuge tube. After a washing step by using 5 ml of 1X Red Blood Cell Lysis Buffer (Abcam, ab204733) for optimal lysis of erythrocytes in a single-cell suspension and after two additional washing steps (1X sterile PBS, 1600 rpm for 10 min), the isolated PBMCs fraction was counted by using LUNA-II Automated Cell Counter (Logos Biosystems).

### DNA extraction from isolated lymphocytes

DNA was isolated from lymphocytes using the AllPrep DNA/RNA Mini Kit (Qiagen, Cat: 80204) according to the manufacturer’s protocol. To enhance DNA concentration, it was eluted twice with 50 μl of Buffer EB following a 1-minute incubation at room temperature. The amount and purity of the extracted DNA were assessed using spectrophotometry and by measuring the A260/280 ratio, which ranged between 1.9 and 2.1, indicating high-quality DNA suitable for subsequent analyses.

### Targeted DNA methylation sequencing with illumina NGS

DNA was bisulfite-converted using the EZ-96 DNA Methylation MagPrep kit (D5041, Zymo Research), followed by two stages of polymerase chain reaction (PCR). In the first round of PCR, primers, including an anchoring sequence targeted at a specific region of the *ELOVL2* gene, were used (Bio-Rad C1000 Touch Thermal Cycler, Bio-Rad Laboratories, CA, USA). Primer sequences are available upon request. Five microliters from the first PCR reaction were then used in a second round of PCR to amplify and barcode the samples using indexing primers. The PCR products were pooled, purified twice using AMPure XP Beads (Beckman Coulter Life Sciences, CA, USA), and quantified via real-time PCR (NEBNext® Library Quant Kit for Illumina, New England Biolabs, MA, USA). The barcoded libraries were sequenced on the Illumina platform using a MiSeq Reagent Nano Kit V2 with a 250 × 2 paired-end sequencing protocol (Illumina, CA, USA).

For data processing, raw paired-end reads were trimmed of sequencing adapters and low-quality sequences using Trim-galore (parameters: trim_galore –illumina –paired –fastqc; available at https://zenodo.org/record/5127899#.Y7RxfOzMJqs). Cleaned data were aligned to the *ELOVL2* reference genome using Bismark [19], and reads deduplicated using UMIs in the forward primers to minimize PCR amplification bias (deduplicate_bismark --paired --barcode –bam). Methylation levels at each CpG site were determined using the Bismark methylation extractor (bismark_methylation_extractor --p --bedGraph --counts --scaffolds --no_overlap), with a minimum threshold of 100 reads per gene for inclusion in the analysis.

### DNA methylation data processing and epigenetic age calculation

IDAT files were processed using the champ.load function from the ChAMP R package [20, 21]. Initial beta values were computed directly from the loaded data to represent methylation levels before normalization.

Following initial data processing, we applied BMIQ (Beta Mixture Quantile dilation) normalization to adjust the beta values. This normalization technique specifically corrects for the technical variation between type I and type II probes by aligning the distribution of type II probe beta values to match those of type I probes. This step ensures that the methylation data are more statistically reliable and comparable across different probe types [22]. Epigenetic age (EpiAge) was calculated for each individual both before and after normalization using the EpiAgePublic algorithm.

### Statistical analysis

For multilinear regression analysis, we utilized Python’s statsmodels library to fit an Ordinary Least Squares (OLS) model. This model was employed to examine the relationship between epigenetic age and several predictors, including cell composition and sex. Our analysis encompassed data preparation, model fitting, and the extraction of key statistics, such as coefficients and p-values.

In addition to multilinear regression, our study also involved other statistical techniques to address specific research questions:

T-tests and Mann-Whitney U tests were used to compare epigenetic ages across different conditions and cohorts, ensuring that we appropriately addressed the distribution characteristics of the data.

Receiver Operating Characteristic (ROC) analysis was conducted to assess the discriminatory power of various epigenetic clocks in distinguishing between different clinical conditions.

ANOVA was utilized to analyze the impact of clinical interventions on epigenetic age across different treatment groups. Statistical tests applied in each figure are described in the figure legend.

## RESULTS

### Development of the EpiAgePublic model across blood and saliva samples

The *ELOVL2* gene has been consistently linked to chronological age across various studies [2]. Analysis of 20 public datasets derived from whole blood using the Illumina BeadChip technology identified *ELOVL2* and *FHL2* as showing the highest correlation between age and DNA methylation [23].

Our research focused on three CpG sites within the *ELOVL2* gene: cg16867657, cg21572722, and cg24724428. Notably, cg16867657 has been recognized as a key age-associated marker with a robust pattern of age-related methylation changes [24]. This was supported by a study examining 421 individuals, which found cg16867657 showing a strong positive correlation with age among 137993 sites (R = 0.957, P-value = 1.20e-228) [25]. The proximity of cg21572722 and cg24724428 to cg16867657, coupled with their known associations with aging (Bell et al., 2012; Christiansen et al., 2016; Hao et al., 2021; Horvath & Raj, 2018; Li et al., 2022; Marioni et al., 2015), justified their inclusion in our EpiAgePublic model. The development of the EpiAgePublic model utilized data from four specific datasets: GSE55763, GSE157131, GSE40279, and GSE30870 (Table 1). The detailed methodology and analysis for model development are outlined in the Methods section of our study.

### Comparative analysis of epiAgePublic with established epigenetic clocks

**Figure 1:**
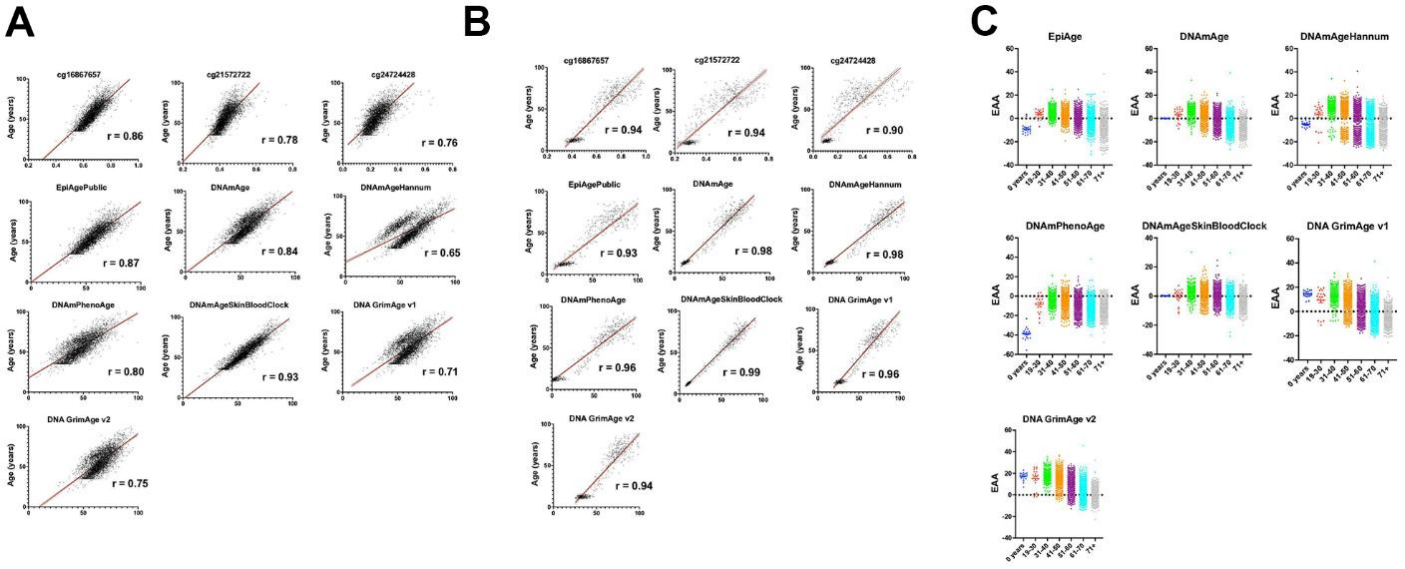
**Comprehensive analysis of epigenetic aging across diverse datasets and demographics.** (**A**) This figure illustrates the correlation between chronological age (y-axis) and measures of epigenetic age including EpiAge, DNAmAge, DNAmAgeHannum, DNAmPhenoAge, DNAmAgeSkinBloodClock, DNAGrimAge v1, and DNAGrimAge v2 (x-axis), as well as individual CpG sites cg16867657, cg21572722, and cg24724428. Data were aggregated from the datasets GSE55763, GSE157131, GSE40279, and GSE30870 (refer to Table 1), encompassing 4625 individuals with ages ranging from 0 to 103 years. The cohort exhibits a rich demographic diversity, including Caucasian-European, Hispanic Mexican, and African American ethnicities, comprising 2506 males, 2079 females, and 40 individuals with unspecified gender. The correlations were assessed using the Pearson r correlation coefficient, denoted by ‘R’ on each plot, highlighting the linear relationship between chronological and epigenetic age across the datasets. All plots achieved a significant p-value of < 0.0001, indicating a strong and statistically significant correlation. Visualization includes a solid black line representing the mean correlation and flanking red lines depicting the 95% confidence interval, illustrating the precision of the correlation estimates and the degree of agreement between chronological and epigenetic age measures across the studied population. (**B**) This figure presents the correlation between chronological age (y-axis) and various measures of epigenetic age (x-axis), including EpiAge, DNAmAge, DNAmAgeHannum, DNAmPhenoAge, DNAmAgeSkinBloodClock, DNAGrimAge v1, and DNAGrimAge v2, alongside individual CpG sites cg16867657, cg21572722, and cg24724428. The data are derived from saliva samples collected from 609 healthy individuals aged 9 to 91 years, detailed in datasets GSE78874, GSE150643, GSE92767, and GSE99029 (referenced in Table 1). The study population includes 310 males and 294 females from diverse ethnic backgrounds—Hispanic, Caucasian, African, and Asian. Correlations are quantified using the Pearson r coefficient, denoted by ‘R’ on each plot, signifying the linear relationship between the two age measures. All correlations are marked by a significance level of p < 0.0001. Visuals include a solid black line indicating the average correlation and red lines showing the 95% confidence interval, emphasizing the reliability and consistency of epigenetic age measures with chronological age across the cohorts. (**C**) This figure presents scatter plots comparing Epigenetic Age Acceleration (EAA) across various age groups. Each dot represents an individual’s EAA value, plotted against their chronological age group. The age groups are categorized as 0 years, 19-30, 31-40, 41-50, 51-60, 61-70, and 71+ years. The vertical axis indicates the EAA, while the horizontal axis delineates the age groups. A horizontal line at zero on the plot marks the threshold between age acceleration and deceleration; points above this line indicate epigenetic age acceleration, while points below indicate deceleration. This visualization highlights trends and patterns in EAA across the lifespan, offering insights into how biological aging progresses relative to chronological aging across different stages of life.

We conducted comparative analyses to evaluate the EpiAgePublic model’s performance relative to established epigenetic clocks. This analysis utilized the same datasets originally employed for the development of the EpiAgePublic model: GSE55763, GSE157131, GSE40279, and GSE30870, all derived from blood samples. This included comparisons of cg16867657, cg21572722, and cg24724428, as well as the EpiAgePublic model, against established epigenetic age models such as DNAmAge (Horvath’s Clock) [26], DNAmAgeHannum [27], DNAmPhenoAge [28], DNAmAgeSkinBloodClock [29], as well as DNA methylation GrimAge versions 1 (DNAGrimAge v1) [30] and 2 (DNAGrimAge v2) [31] (Figure 1A).

The analysis revealed high correlation coefficients for all models. The EpiAgePublic model, which integrates the three CpG sites, exhibited a correlation of 0.87, surpassing individual CpGs (cg16867657, cg21572722, and cg24724428) and closely following DNAmAgeSkinBloodClock, which scored the highest with 0.93. Other clocks displayed slightly lower correlations, with DNAmAgeHannum showing the lowest at 0.65 (Figure 1A).

### Assessing the impact of cell composition and sex on epigenetic age predictions

Our next objective was to determine whether epigenetic age predictions could be affected by blood cell composition and the individual’s sex. To precisely assess the potential confounding effects and correct for variations related to blood cell composition (CD8T cells, CD4T cells, NK cells, B cells, monocytes (Mono), and granulocytes (Gran)), which are inferred from DNA methylation patterns indicative of these cells’ presence [32], we executed multilinear regression analyses adding these confounders as covariates. This detailed examination focused individually on the cg16867657, cg21572722, and cg24724428 CpG sites within the *ELOVL2* gene, as well as collectively within the context of the EpiAgePublic model, comparing these values against DNAmAge (Horvath’s Clock), DNAmAgeHannum, DNAmPhenoAge, DNAmAgeSkinBloodClock, DNAGrimAge v1, and DNAGrimAge v2. These analyses validated the significant and consistent predictive value of chronological age across all individual CpG sites and the EpiAge, even when these confounders are included in the regression (Supplementary Table 1).

Intriguingly, a distinct feature of EpiAgePublic emerged during our analysis: both the individual CpG site assessments and the aggregated EpiAgePublic model demonstrated no significant correlation with sex, exhibiting a P-value of 0.182. This finding contrasts with other epigenetic clocks, where sex showed a significant influence with a P-value of less than 0.05. Specifically, the regression analysis for EpiAgePublic indicated a sex coefficient of 0.388 (std err: 0.291), which was not statistically significant (P>|t|: 0.182), diverging from the trends observed in other epigenetic clocks where the P-value for sex was effectively 0, signifying a highly statistically significant correlation. This distinctive attribute of EpiAgePublic underscores its robustness by highlighting its ability to provide age predictions that are unbiased by sex differences.

### Validating epiAgePublic on saliva samples

Following the development of the EpiAgePublic model, we aimed to validate its applicability to saliva, a noninvasive biological matrix different from blood. The rationale was based on the distinct cellular compositions of saliva, which differ significantly between children and adults in terms of epithelial and immune cells.

Using datasets from GSE78874, GSE150643, GSE92767, and GSE99029, we processed samples from 609 healthy individuals ranging from 9 to 91 years, including 310 males and 294 females from diverse ethnic backgrounds such as Hispanic, Caucasian, African, and Asian using Illumina 450K and Epic array platforms (Table 1).

The comparative analysis of EpiAgePublic and other clocks demonstrated EpiAge’s strong predictive relationship with chronological age, achieving a correlation coefficient of 0.93. DNAmAgeSkinBloodClock exhibited the highest correlation at 0.99 (Figure 1B). These findings validate the utility of EpiAgePublic for non-invasive biological age estimation across different tissues, including saliva, which has obvious advantages as far as accessibility and compliance over blood.

### Age-related epigenetic deceleration across the human lifespan

Our comprehensive dataset encompassed a diverse range of 4,625 individuals, spanning the entire human lifespan from birth to 103 years. The study of Epigenetic Age Acceleration (EAA) traditionally focuses on its clinical significance, often in relation to the acceleration or deceleration of aging and its associated health impacts. Considering that age acceleration may be influenced by various factors such as lifestyle, stress, and socioeconomic conditions, which vary across different age groups, we categorized the dataset into specific age brackets: Newborns, Young Adults (19-30 years), Thirties (31-40 years), Forties (41-50 years), Fifties (51-60 years), Sixties (61-70 years), and Seventies and Beyond (Figure 1C).

To analyze aging acceleration/deceleration patterns within each category, we examined all the epigenetic clocks, consistently observing a trend of epigenetic age deceleration with increasing chronological age among the majority of participants (Figure 1C). Further exploring this phenomenon, we calculated the Pearson r correlation between chronological age and EAA for each clock, assessing the strength and direction of their relationships.

The analysis revealed negative correlations across all clocks, indicating that the rate of epigenetic aging decreases as chronological age increases. Specifically, DNA GrimAge v1 and DNA GrimAge v2 displayed the most significant negative correlations with chronological age (r=-0.6389 and r=-0.6697, respectively), with tight 95% confidence intervals of -0.6556 to -0.6215 and -0.6853 to -0.6535, respectively, underscoring a strong inverse relationship. Other clocks also showed significant negative correlations, as follows: DNAmAge r=-0.5943 (CI: -0.6126 to -0.5753), EpiAgePublic r=-0.4988 (CI: -0.5201 to -0.4768), DNAmAgeHannum r=-0.4424 (CI: -0.4653 to -0.4189), DNAmAgeSkinBloodClock r=-0.4420 (CI: -0.4649 to -0.4185), and DNAmPhenoAge r=-0.3220 (CI: -0.3476 to -0.2959). These highly significant Pearson r correlations (P<0.0001 for all clocks) confirm a consistent inverse relationship between chronological age and EAA, reinforced by a substantial sample size (N=4625 for each clock).

These findings not only corroborate the anticipated pattern of biological aging but also highlight the effectiveness of EpiAgePublic, based on the single gene *ELOVL2*, compared to other clocks that incorporate hundreds or even thousands of CpG sites, as biomarkers for the aging process. The observed age-related trends across the lifespan provide insights into the complex interplay between biological and chronological aging.

### Comparing EpiAgePublic to other epigenetic clocks across cohorts in the context of HIV-related accelerated aging

Early research has indicated a link between HIV and accelerated epigenetic aging, suggesting that HIV-positive individuals may exhibit an advanced biological age [33]. Inspired by findings that demonstrated accelerated aging due to CMV infection using the EpiAge model based on Elovl2 regions in healthy older adults [18], we aimed to explore whether similar patterns could be observed with HIV. We examined the impact of HIV on epigenetic aging using four distinct cohorts: GSE53840, GSE67751, GSE117859, and GSE185391. Our objective was to assess whether the EpiAge model shows accelerated aging in HIV-positive individuals and to compare its performance with other established epigenetic clocks.

We compared EpiAge alongside other epigenetic clocks, including DNAmAge (Horvath’s Clock), DNAmAgeHannum, DNAmPhenoAge, DNAmAgeSkinBloodClock, DNAGrimAge v1, and DNAGrimAge v2. Our analysis employed an unpaired parametric t-test, which passed the normality test, as most data points are normally distributed. Specifically, we compared HIV-positive individuals from the cohorts GSE53840, GSE67751, GSE117859, and GSE185391 against HIV-negative controls from GSE67751. Within the GSE185391 cohort, our focus was on one-time point w0, marking the week immediately preceding the commencement of HIV treatment.

Our comprehensive analysis across a spectrum of epigenetic clocks revealed a significant impact of HIV infection on epigenetic aging. Each clock offers a distinctive view of the aging acceleration that accompanies HIV. Notably, EpiAge displayed pronounced acceleration in epigenetic age among HIV-positive individuals compared to HIV-negative controls, with an average age advancement of 12.04 years (P<0.0001), strongly suggesting a link between HIV and expedited epigenetic aging. Similarly, DNAmAge (Horvath’s Clock) and DNAmAgeHannum showed significant age acceleration in HIV-positive subjects, with increases of 6.612 years (P<0.0001) and 6.230 years (P<0.0001), respectively. The DNAmPhenoAge clock indicated a significant acceleration of 12.79 years (P<0.0001).

**Figure 2:**
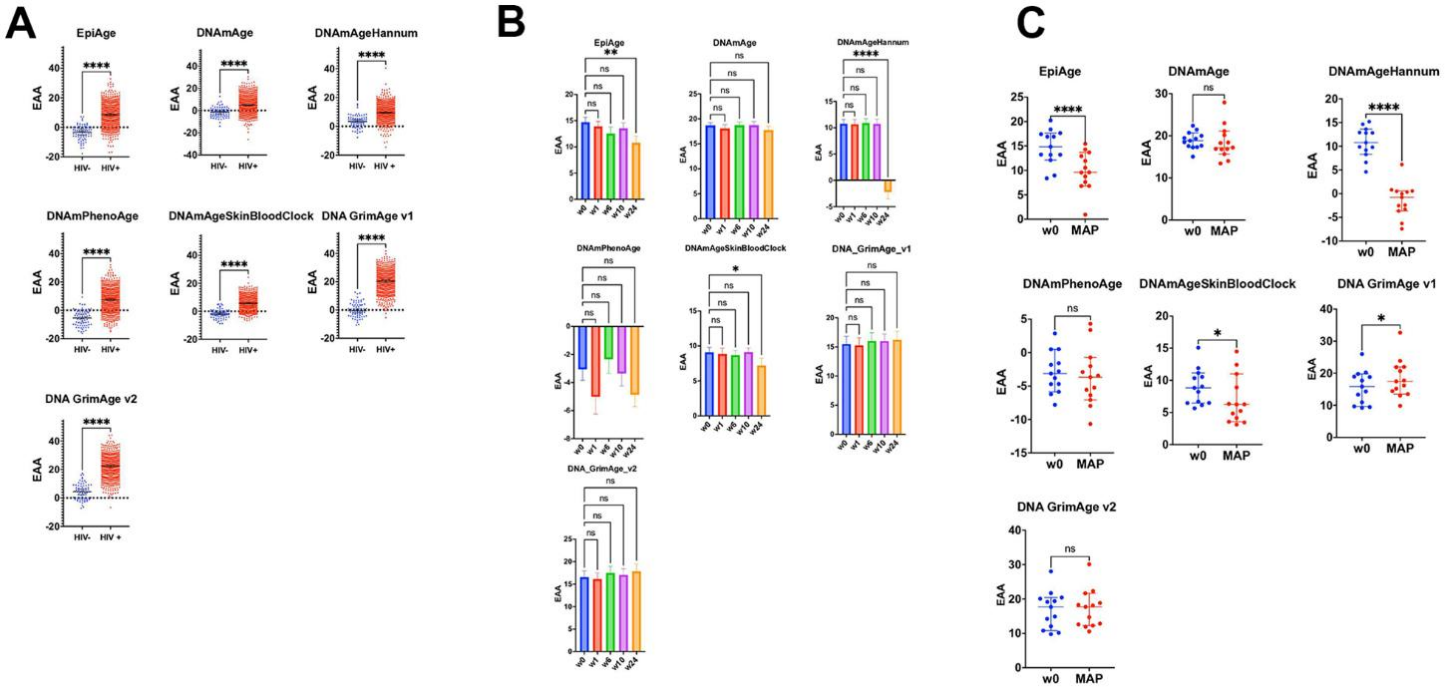
**Impact of HIV on epigenetic age acceleration across multiple cohorts and treatment Phases.** (**A**) Scatter plots comparing epigenetic age acceleration (EAA) across multiple epigenetic clocks: EpiAge, DNAmAge, DNAmAgeHannum, DNAmPhenoAge, DNAmAgeSkinBloodClock, DNAGrimAge v1, and DNAGrimAge v2, between HIV-negative individuals from the GSE67751 cohort (n=69) and HIV-positive individuals from cohorts GSE67751 (n=23), GSE117859 (n=609), GSE53840 (n=120), and GSE185391 (n=86). The plots display median EAA values, with error bars denoting 95% Confidence Intervals (CI). The significance of differences in EAA was tested using unpaired, two-tailed t-tests. A horizontal line at zero on the plot marks the threshold between age acceleration and deceleration; points above this line indicate epigenetic age acceleration, while points below indicate deceleration. (**B**) These scatter plots track changes in EAA from baseline (week 0) through weeks 1, 6, 10, and 24 of a combined HIV treatment strategy, which includes therapeutic vaccination and a latency-reversing agent using multiple epigenetic clocks. We employed repeated measures ANOVA for the analysis. To address potential deviations from the assumption of sphericity, we applied the Geisser-Greenhouse correction as determined by Mauchly’s test. This correction ensures accurate and reliable results in repeated measures analysis, particularly when the equality of variances across the differences between all pairs of groups is not met. (**C**) These scatter plots focus on comparing epigenetic age acceleration (EAA) from baseline (week 0) to the monitored antiretroviral pause (MAP) phase of the BCN02 clinical trial, using various epigenetic clocks: EpiAge, DNAmAge, DNAmAgeHannum, DNAmPhenoAge, DNAmAgeSkinBloodClock, DNAGrimAge v1, and DNAGrimAge v2. Differences were assessed using paired two-tailed t-tests, chosen for their appropriateness given the normal distribution of data.

Further analysis highlighted that the DNAmAgeSkinBloodClock and DNAGrimAge v1 also demonstrated significant aging acceleration, with observed differences of 7.664 years (P<0.0001) and an exceptional 19.97 years (P<0.0001), respectively. These findings are consistent with the hypothesis that HIV infection accelerates biological aging. The consistency of epigenetic clocks, including EpiAge, in revealing accelerated aging in HIV-positive individuals further supports this hypothesis (Figure 2A).

### Assessing the discriminatory power of EpiAge and other epigenetic clocks for HIV status using ROC analysis

To further assess the discriminatory power of EpiAge and other epigenetic clocks in distinguishing between HIV-negative and HIV-positive individuals, we conducted a Receiver Operating Characteristic (ROC) analysis. This method provides a nuanced understanding of each clock’s ability to accurately classify individuals based on their HIV status beyond the significance revealed by t-tests.

In our ROC analysis, EpiAge demonstrated an impressive Area Under the Curve (AUC) of 0.9109, with a sensitivity of 56.37% and specificity of 100% (P<0.0001), indicating appreciable discriminatory power. This suggests that EpiAge is highly capable of differentiating between the two groups based on epigenetic aging patterns.

By comparison, DNAmAge showed an AUC of 0.7835, with a sensitivity of 14.57%, but maintained the same specificity of 100% (P<0.0001), indicating moderate discriminatory ability. DNAmAgeHannum improved upon DNAmAge’s performance, achieving an AUC of 0.8275 with a sensitivity of 14.44% and maintaining 100% specificity (P<0.0001).

DNAmPhenoAge demonstrated strong performance with an AUC of 0.9041, sensitivity of 40.40%, and specificity of 100% (P<0.0001), nearly matching EpiAge’s discriminatory capacity. DNAmAgeSkinBloodClock exhibited a slightly higher AUC of 0.9168, with a sensitivity of 54.30% and perfect specificity (P<0.0001).

Remarkably, DNAGrimAge v1 displayed the highest AUC among the clocks at 0.9795, with an impressive sensitivity of 82.39% and specificity of 100% (P<0.0001), indicating exceptional performance in discriminating between HIV-negative and HIV-positive statuses. DNAGrimAge v2 also showed strong results with an AUC of 0.9595, sensitivity of 72.54%, and unchanged specificity (P<0.0001) (Figure 2A).

### Influence of an HIV treatment strategy on epigenetic aging

Building on these findings, we explored the dynamics of epigenetic aging among HIV-positive individuals to assess how a specific treatment strategy influenced these patterns.

We evaluated the impact of a combined HIV treatment strategy—comprising therapeutic HIV-1 vaccination and the latency-reversing agent romidepsin —using EpiAge, DNAmAge (Horvath’s Clock), DNAmAgeHannum, DNAmPhenoAge, DNAmAgeSkinBloodClock, DNAGrimAge v1, and DNAGrimAge v2. This regimen also incorporated a monitored pause in antiretroviral therapy (MAP), as outlined by Oriol-Tordera et al. [34]. We tracked epigenetic aging from baseline (week 0, before treatment initiation) through several key intervals: weeks 1, 6, 10, and 24, as shown in Figure 2B.

EpiAge uniquely responded to the treatment, showing a significant deceleration in epigenetic aging by week 24, with an adjusted P-value of 0.0061, corresponding to an epigenetic age reduction of approximately 3.93 years from baseline.

DNAmAgeHannum also demonstrated a notable response by week 24, revealing a significant reduction in epigenetic age of 12.96 years (adjusted P-value < 0.0001). DNAmAgeSkinBloodClock displayed a moderate but significant deceleration of 1.88 years over the same period (adjusted P-value = 0.0215). The responses of other clocks did not reach statistical significance (Figure 2B).

Further, we specifically assessed changes from baseline to the Monitored Antiretroviral Pause (MAP) phase, employing paired t-tests to determine if the observed changes in epigenetic age were directly induced by the treatment regimen. This analysis, illustrated in Figure 2C, focused on the unique impacts of the therapeutic vaccination and latency-reversing agent during the pause in antiretroviral therapy. Unlike the continuous treatment period captured in Figure 2B, the MAP phase analysis aimed to isolate the effects of the intervention components without the confounding influence of ongoing antiretroviral therapy (ART).

Among the clocks evaluated, EpiAge and DNAmAgeHannum showed marked responses to the combined therapy despite the MAP. EpiAge demonstrated a significant deceleration in epigenetic aging with a mean difference of -4.811 years (P < 0.0001), while DNAmAgeHannum also indicated substantial deceleration with a mean difference of -12.23 years (P < 0.0001). DNAmAgeSkinBloodClock responded more modestly, showing a deceleration with a mean difference of -2.036 years (P = 0.0129). Surprisingly, DNAGrimAge v1 indicated an unexpected significant acceleration of aging, with a mean difference of +2.628 years (P < 0.0001).

These results underscore the distinct responses of epigenetic clocks to the HIV treatment regimen. EpiAge and DNAmAgeHannum, in particular, stood out for their sensitivity to treatment-induced changes, offering potential as reliable markers for assessing the impact of HIV therapies on biological aging.

### Impact of COVID-19 on epigenetic aging: comparative analysis across multiple epigenetic clocks

**Figure 3:**
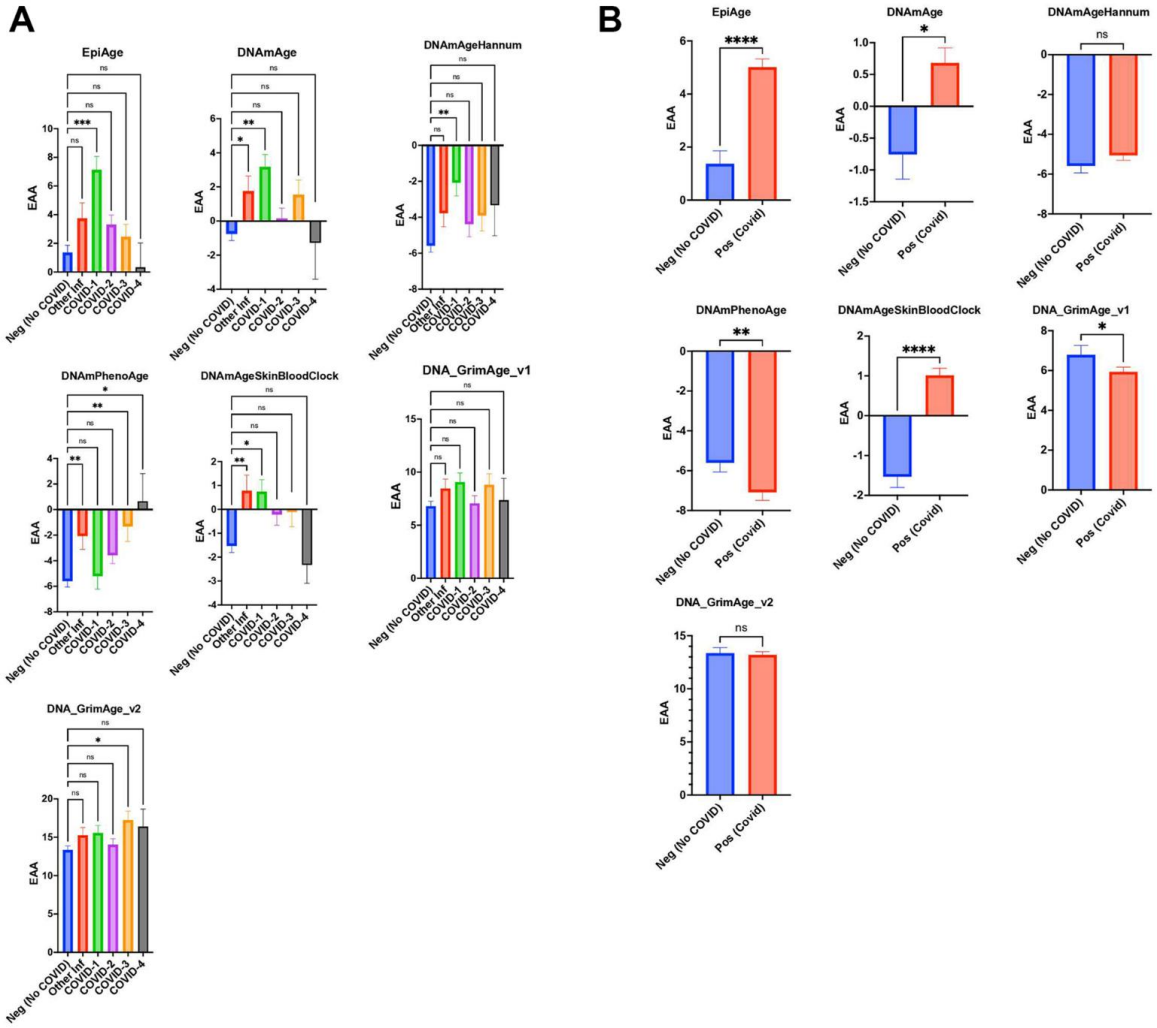
(**A**) Scatter plot analysis of epigenetic age acceleration (EAA) across individuals without COVID-19 (Neg, n=296) from GSE167202 and COVID-19 patients (Pos, n=407) from GSE168739. EAA is calculated by subtracting chronological age from EpiAge estimates. Median EAA values for each group are plotted. Non-parametric two-tailed Mann-Whitney U test reveals significant EAA in COVID-19 patients compared to non-COVID-19 individuals (P < 0.0001), with a median difference of 3.974 years, indicating accelerated biological aging in infected patients. (**B**) Scatter plot analysis comparing epigenetic age between healthy controls (GSE167202, n=296) and COVID-19 patients (GSE168739, n=407), utilizing clocks including EpiAge, DNAmAge, DNAmAgeHannum, DNAmPhenoAge, DNAmAgeSkinBloodClock, DNAGrimAge v1, and DNAGrimAge v2. Due to the non-normal distribution of data, a Mann-Whitney U test was employed. Significance levels are denoted as ns (not significant), * (p < 0.05), ** (p < 0.01), *** (p < 0.001), **** (p < 0.0001).

Our findings indicated that the EpiAge clock showed an age acceleration that varied as a function of the COVID-19 severity score. As such, in our analysis of DNA methylation data from the GSE167202 dataset [35], we found that the EpiAge clock demonstrated a significant age acceleration in COVID-19 severity score 1 compared to negative controls (P=0.0002), with an acceleration of 5.766 years (Figure 3A). Lesser or no significant differences were observed in higher severity scores or in response to an infection by other viruses.

Similarly, DNAmAge indicated significant age acceleration in patients with other infections and mild COVID-19 cases, with age accelerations of 2.514 years (P=0.0166) and 3.935 years (P=0.0035), respectively. DNAmAgeSkinBloodClock mirrored this pattern, showing significant age accelerations for other infections (2.312 years, P=0.0010) and mild COVID-19 severity (2.268 years, P=0.0225), while no significant changes were observed for higher severity levels.

Hannum’s clock only found COVID-19 severity to be significantly associated with age acceleration (3.506 years, P=0.0064), with other severity levels and infections showing no significant differences. PhenoAge displayed significant acceleration for other infections (3.535 years, P=0.0031) and remarkably for COVID-19 severity levels 3 (4.284 years, P=0.0059) and 4 (6.249 years, P=0.0205), but not for level 1.

GrimAge v1 did not show significant differences across groups. GrimAge v2 revealed that COVID-3 severity had a significant age acceleration (3.892 years, P=0.0402) compared to negative control, indicating nuanced responses across different epigenetic clocks to COVID-19 severity and other infections (Figure 3A).

In conjunction with our investigation, we analyzed dataset GSE168739, which was also utilized in a study by Cao et al. [36] This previous study examined the influence of COVID-19 on epigenetic aging in blood samples using a range of established epigenetic clocks, such as Horvath’s DNAmAge, Hannum’s DNAmAge, PhenoAge, SkinBloodClock, and GrimAge. The findings indicated that individuals with COVID-19 experienced a notable increase in DNAm age across all clocks compared to healthy individuals. Extending these observations, our application of the EpiAge clock to these samples demonstrated a significant acceleration in epigenetic aging among COVID-19 patients, with an average advancement of nearly four years (p < 0.0001) (Figure 3B). Similarly, DNAmAge revealed a moderate yet significant age acceleration (p = 0.0114, difference = 1.457 years). The SkinBloodClock also showed significant acceleration (p < 0.0001, difference = 2.648 years), while other clocks displayed no significant changes or, surprisingly, opposite effects such as those seen with GrimAge v1 and PhenoAge.

Based on our analyses, the EpiAge clock demonstrated the most pronounced acceleration in epigenetic aging in response to COVID-19 infection (COVID-19), markedly exceeding the age advancements detected by other clocks. This stark contrast highlights the EpiAge clock’s sensitivity and effectiveness in capturing the impact of COVID-19 on biological aging processes, thus affirming its utility in clinical and epidemiological settings focused on the implications of infectious diseases on aging.

### Current stress, not cumulative stress, is associated with epigenetic age acceleration

To investigate the link between stress and epigenetic age acceleration (EAA), we analyzed whole blood samples from African American individuals using the GSE72680 dataset [37–39].

The original study employed a DNA methylation-based age prediction method, utilizing DNAmAge developed by Horvath [26]. They found that cumulative lifetime stress, as opposed to childhood maltreatment or current stress alone, predicted accelerated epigenetic aging in an urban African American cohort [39].

In our study, we expanded the analysis by using EpiAge, DNAmAge (Horvath’s Clock), DNAmAgeHannum, DNAmPhenoAge, DNAmAgeSkinBloodClock, DNAGrimAge v1, and DNAGrimAge v2. Contrary to the original findings, we did not detect any significant correlation between epigenetic age acceleration and cumulative life stress after correction for blood cell-type composition and lifestyle parameters, such as age, sex, body mass index (BMI), alcohol use, tobacco use, childhood sexual or physical abuse, childhood trauma, cocaine use, heroin use, marijuana use, posttraumatic stress disorder symptom scale, Beck Depression Inventory total score, and treatment for anxiety disorder, bipolar disorder, depression, and posttraumatic stress disorder.

**Figure 4:**
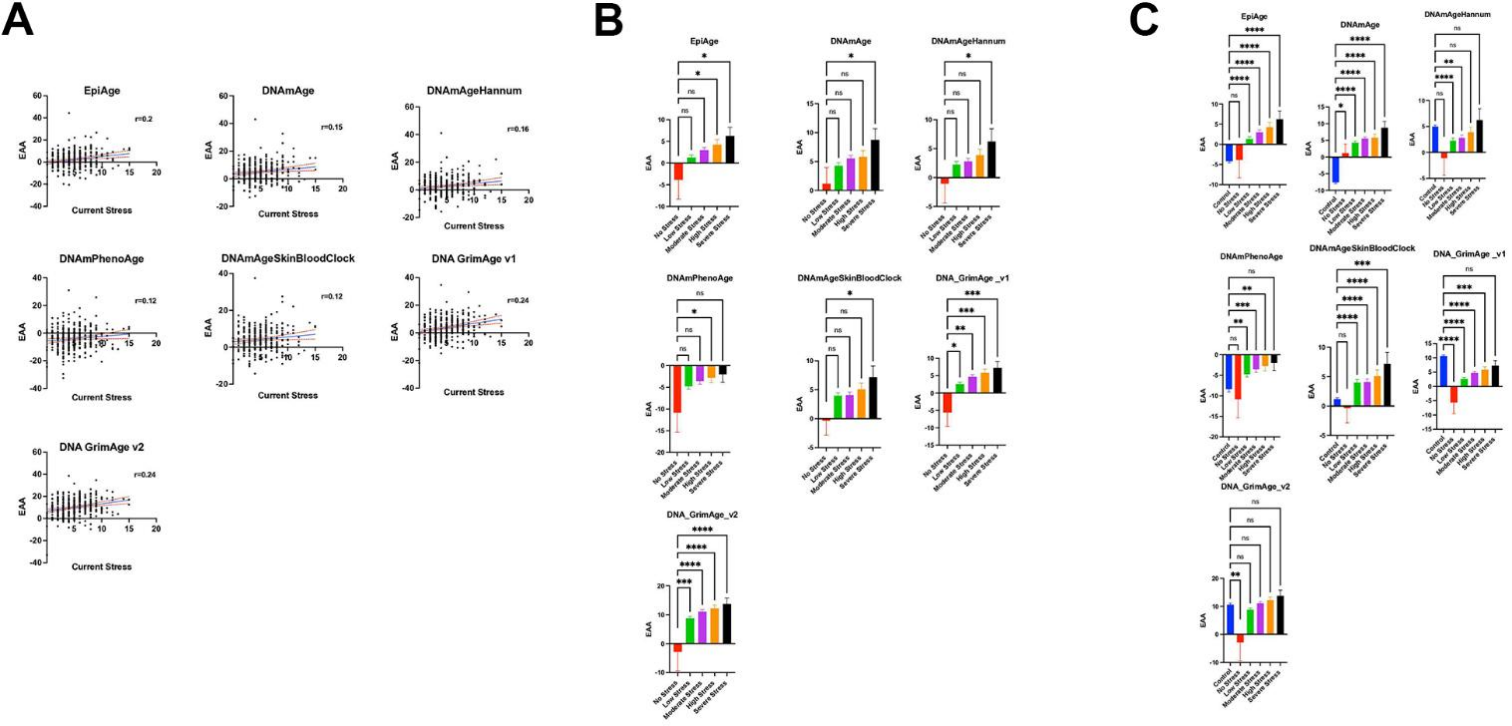
(**A**) Relationship between current stress levels (Axe X) and epigenetic age acceleration (EAA) (axe Y) across various epigenetic clocks (EpiAge, DNAmAge, DNAmAgeHannum, DNAmPhenoAge, DNAmAgeSkinBloodClock, DNAGrimAge v1, and DNAGrimAge v2) from the study GSE72680, analyzed using Pearson’s r. Stress level severity is represented by increasing numbers where a score of 0 means No Stress, and 15 is the most severe stress. Each plot in this figure represents a different clock, highlighting the strength and significance of their relationships with current stress. The central line in each plot represents the regression line, accompanied by two red lines which delineate the 95% confidence intervals, underlining the precision of the correlation estimates. (**B**) Scatter plots illustrate the relationship between varying stress levels and epigenetic age acceleration across different epigenetic clocks. The plot compares five groups categorized by stress intensity: No Stress (Level 0, n=9), Low Stress (Levels 1-4, n=149), Moderate Stress (Levels 5-7, n=106), High Stress (Levels 8-10, n=48), and Severe Stress (Levels 11-15, n=13). Each point represents an individual’s epigenetic age acceleration, with group comparisons analyzed using ordinary one-way ANOVA and Dunnett’s multiple comparisons test to assess statistical differences between the No Stress group and each of the stress intensity groups. (**C**) Scatter plots depicting the relationship between stress levels and epigenetic age acceleration (EAA) across various epigenetic clocks, incorporating control groups for a comprehensive analysis. This plot includes six groups: No Stress (Level 0, n=9), Low Stress (Levels 1-4, n=149), Moderate Stress (Levels 5-7, n=106), High Stress (Levels 8-10, n=48), Severe Stress (Levels 11-15, n=13), and an added Control group (n=419, combining subjects from datasets GSE128235 and GSE125105). Each point illustrates an individual’s EAA. Group differences were statistically evaluated using ordinary one-way ANOVA with Dunnett’s multiple comparisons test, comparing each stress level group as well as the Control group against the No Stress group to identify significant variations in EAA.

Our investigation into the impact of stress on epigenetic age acceleration (EAA) across different epigenetic clocks reveals a complex relationship (Figure 4A). Initially, simple correlations between current stress and various clocks were established, with notable results as follows:

For Horvath’s DNAmAge, a correlation coefficient (r) of 0.1517 and a p-value of 0.0064 were observed. After adjusting for confounders, a significant regression coefficient (β) of 0.5171 was noted, highlighting an association between higher current stress levels and increased EAA (p = 0.030). Cumulative life stress, however, did not exhibit a significant association (p = 0.425).

Hannum’s clock displayed an initial r of 0.1556 and a p-value of 0.0051. Post-correction analysis revealed a β of 0.4831 (p = 0.030), reaffirming the significance of current stress, while cumulative life stress again showed no significant effect (p = 0.399).

PhenoAge presented an r of 0.117 and a p-value of 0.0347 between current stress and EAA. However, the relationship did not remain significant after correction.

DNAmAgeSkinBloodClock demonstrated an initial r of 0.1204 and a p-value of 0.0308 of the association between current stress and EAA, but this significance was not maintained post-correction.

The EpiAge clock showed a stronger initial correlation with current stress (r = 0.1975, p-value of 0.0004), which remained significant after correction (p ≤ 0.037).

For Grimage v1, we found an initial r of 0.2367 with a p-value of <0.0001 association with current stress, which persisted as significant after correction (p = 0.011).

Grimage v2 revealed an r of 0.235 and a p-value of <0.0001, with the association remaining significant post-correction (p = 0.005).

Cumulative life stress did not demonstrate a significant association with EAA across any of the clocks, both before and after applying corrections for confounding factors. This analysis underscores the specific impact of current stress on epigenetic aging, which remained after corrections for blood cell-type composition and lifestyle parameters. These findings highlight the importance of current stress as a significant factor in epigenetic age acceleration across multiple epigenetic clocks.

We further categorized individuals from the GSE72680 dataset [37] into five groups based on the intensity or severity of stress: No Stress (Level 0), Low Stress (Levels 1-4) for mild stress experiences, Moderate Stress (Levels 5-7), High Stress (Levels 8-10), and Severe Stress (Levels 11-15). We examined the association of these stress levels with Epigenetic Age Acceleration (EAA) using the EpiAge clock (Figure 4B). The analysis revealed a gradual increase in EAA from the No Stress Group to the Severe Stress Group. Specifically, we noted a borderline significant difference between the Low Slow-stress and No Stress groups (P = 0.1811) and the Moderate Stress Group (P = 0.0604), a significant difference between the High Stress and No Stress groups (P = 0.0268), and the most pronounced difference was observed between the Severe Stress and No Stress groups (P = 0.0143).

A similar trend was observed across various clocks: For DNAmAge, the comparisons yielded P-values of 0.4334 (Low vs. No Stress), 0.2114 (Moderate vs. No Stress), 0.195 (High vs. No Stress), and 0.0443 (Severe vs. No Stress). Hannum’s clock followed suit with P-values of 0.3713, 0.2711, 0.1466, and 0.0476, respectively. The trend persisted with PhenoAge and DNAmAgeSkinBloodClock, with PhenoAge showing a borderline significant difference at high-stress levels (P=0.0428) compared to no stress and DNAmAgeSkinBloodClock revealing a significant difference at severe stress levels (P=0.0352).

DNA_GrimAge_v1 and v2 showed increasingly significant differences with rising stress levels, underscoring a clear association between stress and epigenetic age acceleration. Specifically, DNA_GrimAge_v1 and v2 displayed significant differences at even low-stress levels (P=0.0110 and P=0.0009, respectively), becoming more pronounced at moderate to severe stress levels, culminating in P<0.0001 for severe stress.

These analyses, conducted using one-way ANOVA with Dunnett’s multiple comparisons test, highlight a consistent trend across all clocks, indicating a significant relationship between stress levels and epigenetic age acceleration.

The GSE72680 dataset had a limited number of 9 samples in the no stress group, which might explain the loss of power to detect EAA differences in the lower stress groups. We therefore included additional control (normal stress) subjects from a different data set GSE128235 (209 subjects) and GSE125105 (210 subjects) datasets in the analysis, as illustrated in Figure 4C. Importantly, there was no significant difference in EpiAge between these newly added control samples and the no-stress group included in the GSE72680 data (Adjusted P-value >0.9999), which suggested that these control groups could be combined in our analysis. When the analysis included a larger sample of “normal” controls, a marked increase in Epigenetic Age Acceleration (EAA) was associated with stress from low to severe stress levels, which was significant even in the low stress group (Adjusted P-value < 0.0001).

For DNAmAge, the no-stress group exhibited significantly higher EAA than the control group (p = 0.0197), with a more pronounced EAA observed across all stress levels in comparison to the control group.

The DNAmAgeHannum analysis indicated a borderline acceleration in EAA within the no-stress group compared to the added control group (Adjusted P-value =0.0720). However, EAA was notably higher in the control group than in both the low and moderate stress groups, with no significant differences noted when compared to the high and severe stress levels.

The PhenoAge analysis aligned with that of EpiAge, showing no distinction between the control and no-stress groups. Yet, there were significant escalations in EAA from low to high-stress levels in comparison to the control group. Intriguingly, PhenoAge suggested an overall deceleration of EAA across all groups, regardless of stress level. Similarly, DNAmAgeSkinBloodClock analysis showed no variance between the control and no-stress groups but revealed significant increases in EAA from low to severe stress levels.

Notably, DNA GrimAge v1 indicated a significantly higher EAA across all groups except for the severe stress group when compared to the control group. DNA GrimAge v2 exhibited significantly higher EAA in the control group than in the no-stress group but did not show marked differences from other stress levels.

Altogether, these findings indicate that EpiAge showed the clearest sensitivity in detecting the stress-induced epigenetic age acceleration, whereby the differences in EAA were only observed between stressed and both controls and non-stressed controls. Furthermore, although all clocks were sensitive to stress, there was observed variability across different epigenetic clocks. These may be attributed to the varied number of CpGs analyzed in the different clocks and the absence of CpGs included in some clocks in the datasets that used different array designs. For example, there was a consistent issue with missing probes in the GSE72680 dataset for DNAmGrimAge2 (ranging typically from 57 to 64 out of 1331 probes) and DNAmFitAge (consistently 48 out of 789 probes). Furthermore, the unexpected results seen with GrimAge v1 and v2 in control groups, derived from diverse studies, may reflect batch effects. It is advised against attempting to correct or process the array data in ways that might lead to the exclusion of CpGs. These observations emphasize the consistency and reliability offered by utilizing fewer CpGs in the EpiAge analysis, which appears to yield more stable results across different conditions.

### Epigenetic age acceleration in Down syndrome

To further assess the value of the EpiAge clock in detecting epigenetic age acceleration in disease states, in this study, we analyzed 22 healthy individuals and 20 individuals with Down syndrome (DS) using the Epic array. Down syndrome is the most common chromosomal anomaly in humans that causes mild to significant developmental, physical, and intellectual disabilities. Due to the trisomy of chromosome 21, which harbours the gene encoding for the amyloid precursor protein (APP), people with Down syndrome progressively develop Alzheimer’s disease neuropathology starting early in life.

**Figure 5:**
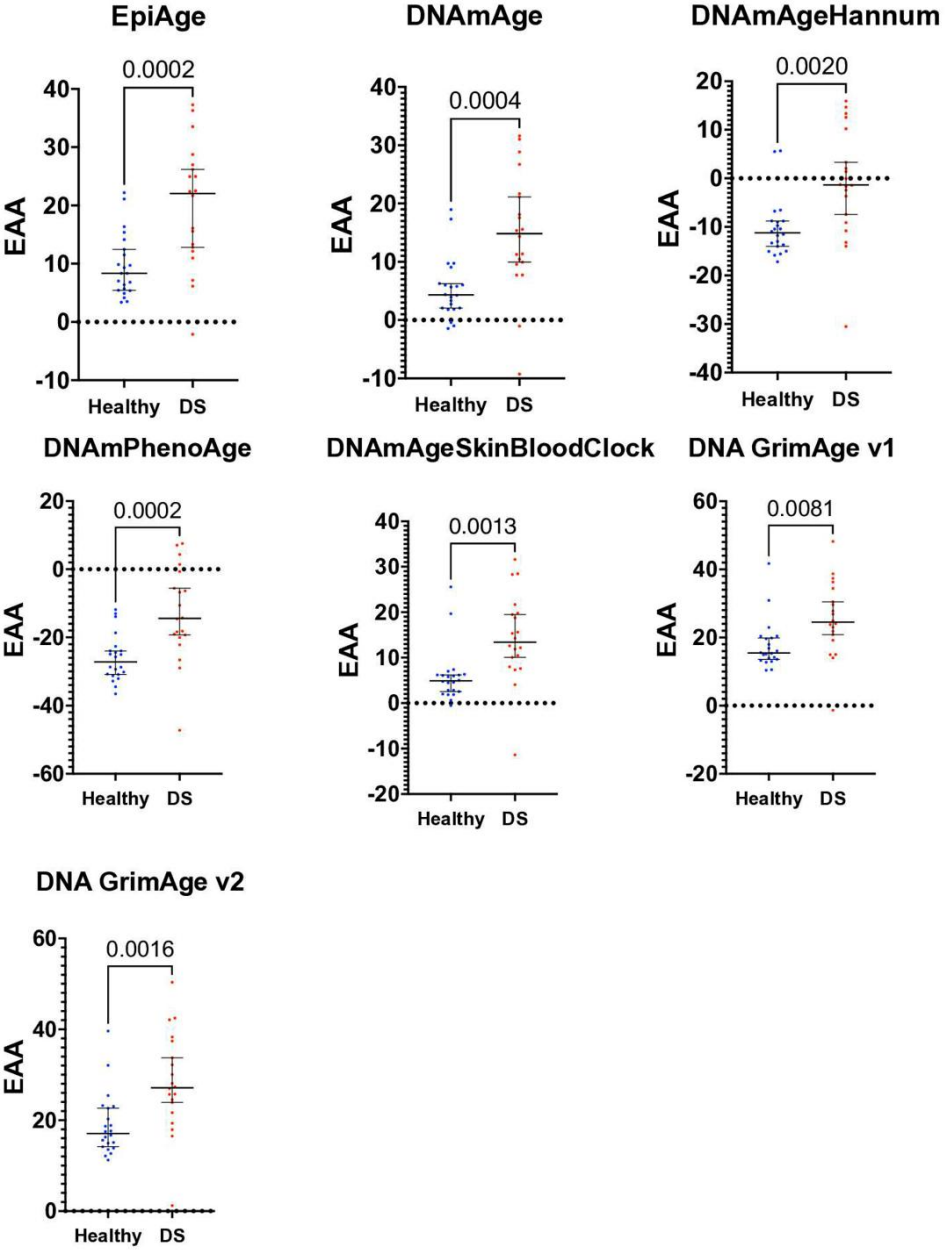
**Scatter plot of epigenetic age acceleration (EAA) in healthy individuals and those with Down syndrome.** This scatter plot illustrates the Epigenetic Age Acceleration (EAA) for each participant in our study, contrasting healthy controls (n=22) with individuals diagnosed with Down syndrome (DS, n=20). EAA, determined by the discrepancy between epigenetic age and chronological age, is plotted for each individual, utilizing various epigenetic clocks: EpiAgePublic, DNAmAge, DNAmAgeHannum, PhenoAge, DNAmAgeSkinBloodClock, GrimAge v1, and GrimAge v2. Each point represents one individual’s EAA, with separate color codes for healthy controls and DS individuals. Unpaired t-tests were conducted to examine the differences in EAA between the groups, showing significant increase in EAA in DS: EpiAgePublic (p=0.0002), DNAmAge (p=0.0004), DNAmAgeHannum (p=0.002), PhenoAge (p=0.0002), DNAmAgeSkinBloodClock (p=0.0013), GrimAge v1 (p=0.0081), and GrimAge v2 (p=0.0016). ROC analysis, discussed in the results section, was performed to further assess the discriminative capability of each clock between the healthy and DS groups. This plot highlights the significant epigenetic age acceleration observed in individuals with Down syndrome in comparison to healthy controls.

The two groups showed no significant chronological age difference, with the mean age for the healthy group at 40.95 and for the Down syndrome group at 40.65, and a p-value of 0.9146 as revealed using an unpaired parametric T-test (applied due to normal distribution). We then calculated epigenetic age using the EpiAgePublic model, among others and assessed epigenetic age acceleration (EAA) (Figure 5A).

Significant age acceleration in the Down syndrome group was observed across all clocks using T-tests and Receiver Operating Characteristic (ROC) analysis to examine the differences. Specifically, for EpiAgePublic, the p-value was 0.0002, with a mean difference ± SEM of 10.26 ± 2.539 and an ROC of 0.8205. DNAmAge showed a p-value of 0.0004, with a mean difference of 9.613 ± 2.481 and an ROC of 0.8341.

DNAmAgeHannum had a p-value of 0.002, with a mean difference of 9.177 ± 2.771 and an ROC of 0.7932. PhenoAge’s p-value was 0.0002, with a mean difference of 13.16 ± 3.272 and an ROC of 0.8318. The DNAmAgeSkinBloodClock had a p-value of 0.0013, with a mean difference of 8.439 ± 2.446 and an ROC of 0.8523. GrimAge v1 had a p-value of 0.0081, with a mean difference of 7.799 ± 2.798 and an ROC of 0.7818, while GrimAge v2 showed a p-value of 0.0016, with a mean difference of 9.404 ± 2.779 and an ROC of 0.8227. (The mean difference ± SEM represents the difference in the mean epigenetic age acceleration (EAA) between the Down syndrome and healthy control groups, along with the standard error of that difference.)

Overall, the EpiAgePublic clock performs comparably to other established epigenetic clocks in detecting age acceleration in individuals with Down syndrome.

### Epigenetic age and Alzheimer’s disease

In all previous analyses, we utilized array technology, which is known for its limitations. This technology requires normalization methods such as BMIQ and others to adjust for variability in methylation data. However, a major concern with array technologies, such as the Illumina Infinium Methylation BeadChip often used in epigenome-wide association studies (EWAS), is the presence of batch effects. These effects can reduce experimental power and potentially lead to false positive results due to variations in the day of processing, the individual glass slide, and the array’s position on the slide. Despite employing batch-effect removal tools like ComBat and Harman, residual batch effects persist and require significant correction [40].

In contrast, targeted next-generation sequencing offers advantages over array technologies, including higher resolution and greater specificity for detecting methylation changes across diverse biological samples. This method is less susceptible to batch effects and does not rely on pre-designed probes, allowing for a more comprehensive analysis of the methylome.

To validate the EpiAge model using targeted NGS, we conducted an observational longitudinal clinical study where we analyzed DNA extracted from the lymphocytes of peripheral blood mononuclear cells (PBMCs) from 55 individuals, categorized into Alzheimer’s disease patients and control participants. One participant was excluded from the analysis due to technical issues, leaving a total of 54 participants. Among these, there were 10 males and 16 females in the control group and 7 males and 21 females in the Alzheimer’s disease (AD) group. The mean age of the control group was 71.53 years (SD = 9.36), while the AD group had a mean age of 72.57 years (SD = 8.89). A parametric t-test showed no significant difference in age between the control and AD groups (p = 0.6793).

For this study, we expanded the region analyzed in the *ELOVL2* gene to include not only the well-established CpG sites—cg16867657, cg21572722, and cg24724428—from the Illumina array but also an additional ten CpG sites, totaling 13 sites. This broader scope was intended to enhance the technical robustness of the EpiAge model.

First, we examined the replication consistency across our samples. Due to limitations in the amount of DNA obtained, only 41 out of the total 54 individuals had sufficient DNA to perform analyses in four replicates.

**Figure 6:**
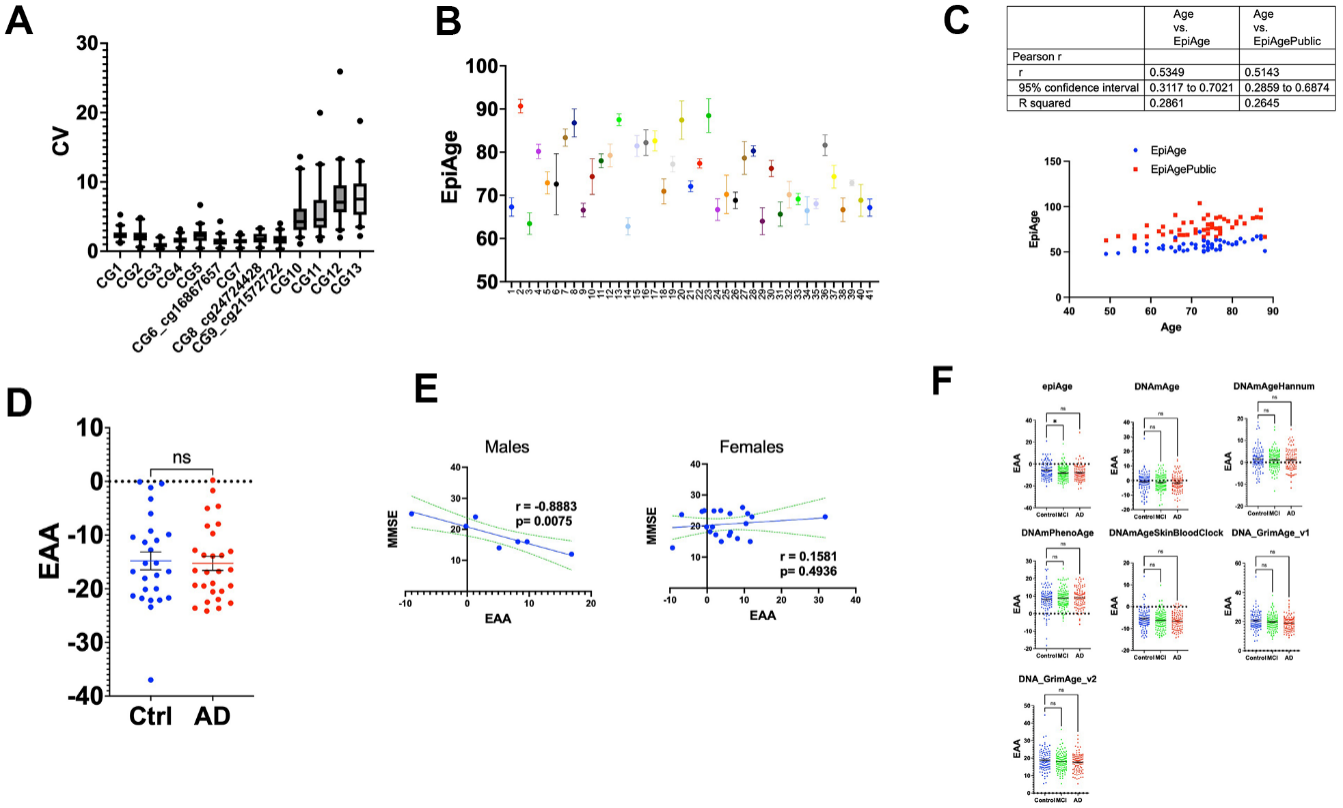
(**A**) Box plots of coefficient of variation (CV) for methylation levels across 13 CpG sites. This figure illustrates the distribution of the coefficient of variation (CV) for each of the 13 CpG sites within the ELOVL2 gene, analyzed using next-generation sequencing. The CV was calculated by dividing the standard deviation of methylation measurements by the mean for each site, expressed as a percentage. The box plots show the interquartile range (25th to 75th percentile) with whiskers extending to the 5th and 95th percentiles. Sites 1-9 exhibit lower CVs, ranging from 0.28% to 6.7%, indicative of high methylation consistency. Sites 10-13 display higher CVs, ranging from 1% to 25%, reflecting increased variability in regions of lower methylation. This variability highlights the influence of methylation levels on the precision of epigenetic age assessments. (**B**) The figure displays a scatter plot of epigenetic age measurements using our newly developed EpiAge next-generation sequencing assay for 41 study participants, using the linear regression model developed for EpiAgePublic in samples that had four technical replicates. Each point on the x-axis corresponds to the average EpiAge calculated for a blood sample (buffy coat) of an individual participant. The y-axis indicates the epigenetic age calculated for each replicate. Error bars represent the 95% confidence intervals for the mean epigenetic age of each individual. (**C**) Comparison analysis between EpiAgePublic (red dots) and EpiAge calculated using the HKG epiTherapeutics proprietary model (blue dots) relative to chronological age (Axe X). Correlation analysis was performed using Pearson’s R. The table provides a comparison of the correlation coefficient (r), 95% confidence interval, and R-squared values. (**D**) Epigenetic Age Acceleration (EAA) Comparison between control and AD patients calculated using EpiAge next-generation sequencing assay. This scatter plot compares the EAA between control participants (n=26) and Alzheimer’s Disease (AD) patients (n=28). Each dot represents an individual, and the plot shows the mean with SEM. The epigenetic age was calculated using the HKG epiTherapeutics proprietary model. Differences in EAA between the groups were analyzed using a two-tailed parametric t-test to assess statistical significance. (**E**) The left panel displays the Pearson correlation between Epigenetic Age Acceleration (EAA) and Mini-Mental State Examination MMSE in 7 male Alzheimer’s patients, while the right panel displays the correlation in 21 female Alzheimer’s patients. The middle line in each plot represents the linear regression fit, while the two lines surrounding it represent the 95% confidence bands, which indicate the variability of the correlation. (**F**) Comparative Analysis of Epigenetic Age Acceleration Across Alzheimer’s Disease, Mild Cognitive Impairment, and Control Groups Using Multiple Epigenetic Clocks. This figure presents the comparison of Epigenetic Age Acceleration (EAA) using multiple clocks: EpiAge, DNAmAge, DNAmAgeHannum, DNAmPhenoAge, DNAmAgeSkinBloodClock, DNAGrimAge v1, and DNAGrimAge v2. The data were derived from 96 control individuals, 111 with mild cognitive impairment (MCI), and 93 with Alzheimer’s Disease from dataset GSE144858, using DNA from human blood. The plot shows means with Standard Error of the Mean (SEM). For statistical analysis, we employed an ordinary one-way ANOVA to compare AD (Alzheimer’s) to controls and MCI to controls. Parametric ANOVA was used due to the normal distribution of the data. ‘Ns’ stands for not significant; * for p < 0.05; **’ for p < 0.01; *** for p < 0.001; and **** for p < 0.0001.

In our analysis, the coefficient of variation (CV) was calculated for each of the 13 CpG sites to evaluate the consistency of methylation measurements obtained via next-generation sequencing. The CV was determined by dividing the standard deviation by the mean methylation level for each site and expressed as a percentage. The results, depicted in box plots (Figure 6A), indicate that the CVs for the first nine CpG sites were relatively low, ranging from 0.28% to 6.7%, as these CpGs exhibited high percentages of methylation. In contrast, the CVs for sites 10 through 13, which are characterized by lower methylation levels (around 10% or less), showed greater variability, ranging from 1% to 25% across individuals. This increased variability at lower methylation levels aligns with statistical expectations, where measurements near the extremes tend to have lower relative variability, while those closer to the midpoint or at lower levels tend to fluctuate more [41, 42].

In our study, we assessed the technical accuracy of EpiAge in a cohort of 41 participants (Figure 6B), each tested four times using targeted next-generation sequencing (NGS). We first evaluated the Confidence Interval (CI) Range as a measure of variability in the epigenetic age estimation across repeated measurements:

CI Range <1: Observed in 14 individuals, suggesting a high precision in epigenetic age estimation for approximately 34% of the participants.

CI Range >1 and <1.5: Found in 15 individuals, indicating moderate variability in epigenetic age estimates, affecting about 37% of the study population.

CI Range >1.5 and <2: Present in 7 individuals, reflecting a greater variability in measurements, impacting around 17% of the participants.

CI Range >2 and <3: Noted in 4 individuals, demonstrating significant variability, which was evident in approximately 10% of the cohort.

CI Range >3: This highest variability was observed in 1 individual, accounting for about 2% of the participants.

Additionally, we summarized key descriptive statistics, including mean, standard deviation (SD), and coefficient of variation (CV) for each participant in Supplementary Table 2. The results showed that the CVs ranged from 0.57% to 6.10%, demonstrating varying levels of technical accuracy.

- These results show that the targeted NGS-based EpiAge exhibited high reliability, with most participants displaying low CI ranges and low coefficients of variation.
- The CVs indicate that the method’s precision varies across individuals but generally falls within acceptable limits for biological measurements.
- NGS offers improved accuracy compared to traditional array-based methods, but some variability remains, potentially due to noise in the replicates.
- The findings highlight the accuracy of EpiAge by NGS, although further optimization could improve consistency and reduce noise in repeated measurements.

These findings demonstrate that while most study participants exhibited relatively low to moderate variability in epigenetic age estimates, a smaller group showed significant variability. This distribution underscores the importance of considering technical variability in such analyses. While targeted next-generation sequencing provides high-resolution and specific detection of methylation changes, there is inherent variability that must be accounted for in clinical and research settings. The analysis of CI Ranges provides important insights into the reliability of epigenetic age measurements and their potential application in longitudinal studies and interventions aimed at aging and related neurodegenerative disorders.

Furthermore, these results highlight the importance of evaluating confidence intervals when interpreting epigenetic age to ensure robust conclusions. This is particularly vital in clinical or research contexts where precision is paramount. Relying solely on single measurements without technical replicates, which is common with less accurate array technologies, overlooks the importance of accuracy and the limitations of study design. Without this consideration, it becomes challenging to discern whether observed changes in epigenetic age across multiple time points are due to actual biological effects, batch effects, or merely technical noise.

We subsequently calculated the epigenetic age using the bisulfite-targeted next-generation sequencing data and the EpiAgePublic model (including 3 CpGs: cg16867657, cg21572722, and cg24724428), which was trained on GSE55763, GSE157131, GSE40279, and GSE30870 (Table 1), as discussed above. We also calculated EpiAge using a proprietary model developed by HKG epiTherapeutics, as discussed in the Methods section, which was originally developed using saliva samples (Figure 6C). A comparison analysis between the two models revealed a slightly better performance for the proprietary model (r = 0.5349) compared to EpiAgePublic (r = 0.5143). The Epigenetic Age Acceleration (EAA) was determined by subtracting the EpiAge value from the proprietary model from the chronological age for 26 control participants and 28 patients with Alzheimer’s Disease (AD). Our analysis revealed no significant difference between the two groups (parametric t-test, p = 0.8188) (Figure 6D).

We further analyzed whether Mini-Mental State Examination (MMSE) is associated with EAA separately in females and males. Interestingly, a strong negative correlation between MMSE T0 and epigenetic age acceleration was observed in males (r = -0.8883, p = 0.0075) (Figure 6D), while no significant correlation was observed in females (Figure 6E).

To examine EAA in AD patients in other studies, we analyzed the Epic Array dataset GSE144858 [43], derived from the EU-funded AddNeuroMed Cohort, a large cross-European AD biomarker study using human blood DNA [44, 45]. This dataset included 96 control individuals, 111 with mild cognitive impairment (MCI), and 93 with Alzheimer’s Disease (AD).

We applied the EpiAgePublic model and other epigenetic clocks (Figure 6E). Consistent with the bisulfite next-generation sequencing EpiAge assay, we did not observe any significant EAA differences between AD and controls, based on the Kruskal-Wallis test, since the data did not pass the normality test. Notably, EpiAgePublic was the only model showing significant age deceleration in MCI compared to controls (adjusted p-value = 0.0339).

## DISCUSSION

This study introduces epiAgePublic, a novel epigenetic aging model utilizing only three CpG sites within the *ELOVL2* gene, traditionally known for its strong association with aging [24, 25].

The simplicity and precision of epiAgePublic, designed for compatibility with next-generation sequencing (NGS) technologies, mark a significant step forward in the field of epigenetic research. Our findings demonstrate that epiAgePublic can effectively estimate biological age with an accuracy comparable to more complex, established epigenetic clocks. This could potentially streamline and reduce the cost of biological age assessments in clinical settings.

In developing the epiAgePublic model, we focused on three CpG sites within the *ELOVL2* gene—cg16867657, cg21572722, and cg24724428—known for their links to aging, supported by a body of research (Bell et al., 2012; Christiansen et al., 2016; Hao et al., 2021; Horvath & Raj, 2018; Li et al., 2022; Marioni et al., 2015). Employing linear regression, we trained a model utilizing a training cohort compiled from four public databases (GSE55763, GSE157131, GSE40279, and GSE30870), capturing a wide demographic range (Table 1). The diversity of this training cohort was crucial for developing an inclusive EpiAgePublic model, which achieved an R-squared value of 0.7512.

The model was validated on several cohorts (Table 1 and Figure 1) and was highly correlated with chronological age. Furthermore, our comparative analysis with established epigenetic clocks revealed EpiAgePublic’s robustness and its ability to assess biological aging. epiAgePublic remains significantly correlated with chronological age even when potential confounding factors like blood cell composition and sex are included in the model. Age consistently emerged as a significant predictor across all models, underscoring its fundamental role in epigenetic aging, while sex exhibited a nuanced influence, revealing subtle but significant associations with all models except epiAgePublic.

The deceleration of epigenetic aging relative to chronological age, as individuals grow older suggests a nuanced interaction between genetics, environment, and aging processes. This phenomenon (Figure 1C) might reflect adaptive mechanisms or biological resilience in response to environmental stressors and lifestyle factors across the lifespan. This deceleration of epigenetic aging in long-livers also aligns with findings from other studies [46].

After developing the EpiAgePublic model, a crucial validation step was undertaken to confirm its utility not just for blood samples but also for saliva, thereby expanding its application range. This validation is pivotal, serving both as an independent test on fresh datasets and as an extension of the model’s utility beyond its initial scope, moreover increasing the feasibility and availability of the test as a tool for monitoring public health without requiring invasive blood draw.

The cellular composition of saliva, as well as blood, could vary across individuals, reflecting varied proportions of epithelial and immune cells. Specifically, saliva in children comprises approximately 35% epithelial cells and 65% immune cells [47], whereas adult saliva contains around 80% epithelial cells and 20% immune cells [48, 49]. Nevertheless, our data show a high correlation across ages between the EpiAge predicted in blood and saliva. This is consistent with previous studies. The *ELOVL2* gene has been consistently identified as a predictive marker of age in saliva samples [50, 51]. The EpiAgePublic model, originally developed for blood, could be effectively applied to saliva, enabling a highly accessible biological tool for measuring biological age.

### Sex confounding effects; EpiAge versus other clocks

Unlike other epigenetic clocks that exhibit a significant correlation with sex, EpiAge’s lack of association with sex may indicate a more refined capacity to isolate the biological essence of aging from sex-based epigenetic variation. This positions EpiAge as a more universally applicable tool for assessing biological age. Therefore, EpiAge could offer a clearer, more focused lens through which to study the aging process, unencumbered by the variation introduced by sex. This advantage underscores the importance of developing and utilizing epigenetic clocks that can accurately reflect the aging process in a manner that is as inclusive and representative as possible of the general population.

Thus, EpiAgePublic, despite being nimble, relying on a single small genomic region is nevertheless an effective measure of the aging process comparable or even superior to other clocks that incorporate hundreds or even thousands of CpG sites.

### Understanding HIV-related accelerated epigenetic aging

The investigation into HIV-related accelerated epigenetic aging represents a critical aspect of our study, shedding light on the intricate relationship between HIV infection and biological aging. Building upon previous research linking HIV to expedited epigenetic aging processes, our study delved into the specific impacts of HIV infection on epigenetic aging using multiple cohorts and compared the performance of various epigenetic clocks, including EpiAge, in elucidating these patterns.

Our findings corroborate earlier observations [52] suggesting a significant association between HIV infection and accelerated epigenetic aging. Notably, EpiAge demonstrated remarkable sensitivity in detecting age acceleration among HIV-positive individuals, with a substantial average advancement of 12.04 years compared to HIV-negative controls. This aligns with previous studies highlighting the accelerated aging phenomenon in HIV-infected populations and underscores the utility of EpiAge as a reliable marker for assessing epigenetic changes associated with HIV infection.

Furthermore, our comparative analysis across multiple epigenetic clocks revealed consistent evidence of aging acceleration in HIV-positive individuals, as indicated by DNAmAge, DNAmAgeHannum, DNAmPhenoAge, DNAmAgeSkinBloodClock, and DNAGrimAge v1. This acceleration is possibly due to inflammatory processes associated with HIV infection and, therefore, might be related to other inflammatory diseases as well. These findings underscore the robustness of epigenetic clocks in capturing the biological consequences of HIV infection on aging processes, thereby providing valuable insights into the pathophysiology of HIV-related accelerated aging.

In addition to identifying accelerated epigenetic aging patterns, our study evaluated the discriminatory power of epigenetic clocks in distinguishing between HIV-negative and HIV-positive individuals. ROC analysis highlighted the superior performance of EpiAge in accurately classifying individuals based on their HIV status, further underscoring its potential as a sensitive biomarker for HIV-related accelerated aging.

Moreover, our investigation into the influence of HIV treatment strategies on epigenetic aging dynamics yielded intriguing results. The observed deceleration in epigenetic aging following specific pharmacological treatment interventions underscores the potential reversibility of age-related epigenetic changes in HIV-positive individuals. Notably, EpiAge and DNAmAgeHannum exhibited significant responses to treatment, suggesting their utility as sensitive indicators of treatment-induced changes in biological aging. An open question remains in future studies to establish the specific effects of the different drug classes used in current antiretroviral therapy on epigenetic aging [33]and their correlation with changes in CD4+ cell counts and the CD4/CD8 ratio as well as with long-term clinical outcomes in individuals with HIV.

Overall, our study provides comprehensive insights into the complex interplay between HIV infection, epigenetic aging, and treatment interventions. By elucidating the underlying mechanisms driving HIV-related accelerated aging and evaluating the efficacy of epigenetic clocks in capturing these dynamics, our findings contribute to the growing body of knowledge aimed at improving clinical outcomes and therapeutic strategies for HIV-infected individuals.

### Impact of COVID-19 on biological aging as measured by the different epigenetic clocks

The relationship between COVID-19 infection and epigenetic age acceleration (EAA) is complex and continues to be a subject of significant scientific debate. While some studies have found no notable epigenetic acceleration in COVID-19 patients [53], others have reported marked changes [36, 54]. Our study shows the effects of COVID-19 on biological aging, with variations in response depending on the severity of the disease and the specific epigenetic clock used.

Different epigenetic clocks, including the EpiAge metric described here, show different sensitivity to changes induced by the virus. This variability can be attributed to the clocks’ distinct molecular foundations and their differential responsiveness to the biological pathways affected by COVID-19.

For instance, the EpiAge metric, designed to capture age-related changes through specific CpG sites within the ELOVL2 gene, detects accelerated aging in less severe COVID-19 cases. This suggests that EpiAge may be particularly sensitive to early biological changes that other clocks might miss. Surprisingly, as COVID-19 severity increased, the EAA did not show significant differences. This could be attributed to severe cases overwhelming the immune system, thereby masking such changes.

Conversely, clocks like DNAmAgeSkinBloodClock and DNAGrimAge, which incorporate a broader array of CpG sites, showed significant changes primarily in cases with higher disease severity, reflecting their potential to capture larger biological disturbances.

Our analysis using multiple epigenetic clocks underscores the complex landscape in which COVID-19 infection and its severity levels affect biological aging. Notably, our findings are in line with other recent studies reporting accelerated epigenetic aging in COVID-19 patients [36], as further evidenced by our analysis of the GSE168739 dataset. These results support the utility of EpiAge and similar metrics in shedding light on the biological consequences of COVID-19, underscoring the importance of epigenetic clocks in understanding the broader implications of this and other infectious diseases on human health and aging. In future long-term observational studies, it would also be interesting to evaluate the potential relationship between Epigenetic Age Acceleration and cognitive dysfunction in individuals with post-COVID-19 syndrome.

### Stress and epigenetic age acceleration

Existing research on the association between stress and epigenetic age acceleration has shown conflicting or inconclusive results [55]. This inconsistency may stem from the selection of different genes in various epigenetic models, which might not be responsive to stress factors. The EpiAge model, which incorporates the *ELOVL2* gene, demonstrates, however, a clear association between current stress and epigenetic age acceleration. This suggests that the choice of stress-responsive genes, like *ELOVL2*, is crucial for accurately assessing the impact of stress on biological aging.

In our analysis of the EpiAge model, we’ve uncovered a noteworthy link between current stress and epigenetic age acceleration. Current stress, arising from immediate and ongoing challenges in an individual’s daily life and reflects the current state of an individual’s personal, professional, and social life, encompassing recent events or situations that are directly impacting their well-being now, shows a unique association with accelerated epigenetic aging. This contrasts with the lack of similar associations with other stress types, like cumulative life stress, which represents the lifelong accumulation of stressors [56], network life stress arising from social network events [57], and personal life stress, which focuses on individual-specific life circumstances [58]. These findings, although counterintuitive, highlight the distinct impact of current, day-to-day stressors on biological aging, as opposed to the more chronic or relational stresses captured by other categories. This indicates a unique impact of current stressors, possibly related to their immediate biological effects and to the possibility that the effect of stress on aging is potentially reversible once the burden of current stress is relieved.

This distinct association between current stress and accelerated epigenetic aging opens new avenues for understanding the biological underpinnings of stress responses. It also raises questions about the reversibility of such epigenetic changes with stress management or resolution. Future research might explore the mechanisms behind this phenomenon and investigate whether interventions targeting current stress can effectively decelerate epigenetic aging. This could have profound implications for stress management strategies and their role in healthy aging and stress-related disorders. Zannas et al. have demonstrated that aging and stress can epigenetically synergize in stress-related disorders such as Major Depressive Disorder (MDD) [37]. However, it is currently unknown whether Epigenetic Age Acceleration occurs in severe depressive phenotypes.

Interestingly, our observation that no significant correlation was found between epigenetic age acceleration and cumulative life stress after adjusting for blood cell-type composition and lifestyle parameters, as previously reported by Zannas et al., [39] is partially supported by the recent research conducted by Poganic et al. [59]. They suggest that the effects of acute stress are more pronounced and directly associated with measures of epigenetic age acceleration. This finding is in line with our results, highlighting the predominant influence of current stress over cumulative stress on epigenetic age acceleration, even after controlling blood cell-type composition and lifestyle parameters.

### Epigenetic age acceleration in Down syndrome: insights, implications, and clinical applications

Epigenetic age acceleration in Down syndrome has been a subject of interest in various studies. Down syndrome, characterized by the presence of all or part of a third copy of chromosome 21, has been associated with accelerated epigenetic aging. Studies demonstrated that trisomy 21 significantly increased the biological age of blood and brain tissue by an average of 6.6 years [60]. Furthermore, it has been noted that age acceleration can be observed in both blood and brain tissue in Down syndrome (Milicic et al., 2022). These findings collectively suggest that Down syndrome is associated with accelerated epigenetic aging, as evidenced by various studies focusing on epigenetic age acceleration in individuals with this condition. Additionally, moderate acceleration of epigenetic aging has been described in Down syndrome in another study (Cypris et al., 2020). Other studies have also found evidence of epigenetic age acceleration in newborns with Down syndrome (Xu et al., 2022). The research highlights the importance of understanding the epigenetic mechanisms underlying aging in Down syndrome and its implications for health outcomes in affected individuals. Research has shown that the use of pan-tissue DNA methylation clocks revealed epigenetic age acceleration in segmental progeroid syndromes like Down syndrome (Ashapkin et al., 2019). Furthermore, recent studies have indicated that epigenetic age acceleration can occur in segmental progeria conditions such as Down syndrome (Lee, 2023).

In our study, we further explored epigenetic age acceleration in individuals with Down syndrome using various epigenetic clocks, including the EpiAgePublic model. Our findings corroborate earlier research indicating that Down syndrome, characterized by an extra copy of chromosome 21, is associated with accelerated biological aging. This acceleration was consistently observed across multiple epigenetic clocks, affirming the robustness of these tools in capturing biological age differences.

The comparable performance of the EpiAgePublic clock with other established models suggests its utility as a simpler, potentially more accessible tool for assessing biological aging in clinical settings. The significant differences in epigenetic age between the Down syndrome and healthy control groups highlight the profound impact of trisomy 21 on aging processes. These insights could inform better management strategies for age-related conditions in individuals with Down syndrome, such as cognitive deficits and the early onset of Alzheimer’s disease.

Our results underscore the relevance of using epigenetic clocks to understand aging in Down syndrome. Continued research into the specific epigenetic changes associated with Down syndrome could lead to targeted interventions that might help mitigate the accelerated aging process, ultimately improving health outcomes. This study supports the growing recognition of epigenetic clocks as valuable tools in aging research and their potential application in improving clinical care for populations with unique aging trajectories like those seen in individuals with Down syndrome.

### Epigenetic age in Alzheimer’s disease: insights from EpiAgePublic and other epigenetic clocks

Differences in DNA methylation have been reported both in blood and brain tissue [44, 45] of Alzheimer’s disease cases compared to healthy controls, especially in gene regions associated with AD pathology, including apolipoprotein E (ApoE) and the amyloid precursor protein (APP) but also in other genes [61–68]. However, the direction of the changes is not consistent across studies [44, 45, 69, 70]. Accordingly, the limited reports on epigenetic age in AD have also yielded inconsistent findings [69], and their utility in detecting AD pathology and cognitive status remains a matter of debate.

In the present study, we calculated the epigenetic age using bisulfite-targeted next-generation sequencing data and the EpiAgePublic model (including 3 CpGs: cg16867657, cg21572722, and cg24724428), which was trained on GSE55763, GSE157131, GSE40279, and GSE30870 (Table 1). The Epigenetic Age Acceleration (EAA) was determined for 26 control participants and 28 patients with AD. Our analysis revealed no significant difference between the two groups (parametric t-test, p = 0.9875) (Figure 6C).

We further analyzed whether the Mini-Mental State Examination (MMSE) score is associated with EAA separately in females and males. Interestingly, a strong negative correlation between MMSE T0 and epigenetic age acceleration was observed in males (r = -0.8883, p = 0.0075) (Figure 6D), while no significant correlation was observed in females (Figure 6D).

To examine EAA in AD patients in other studies, we analyzed the Epic Array dataset GSE144858 (Roubroeks et al., 2020), derived from the EU-funded AddNeuroMed Cohort, a large cross-European AD biomarker study using human blood DNA (Fransquet et al., 2021; Levine et al., 2015). This dataset included 96 control individuals, 111 with mild cognitive impairment (MCI), and 93 with Alzheimer’s Disease (AD).

We applied the EpiAgePublic model and other epigenetic clocks (Figure 6E). Consistent with the bisulfite next-generation sequencing EpiAge assay, we did not observe any significant EAA differences between AD and controls, based on the Kruskal-Wallis test, since the data did not pass the normality test. Notably, EpiAgePublic was the only model showing significant age deceleration in MCI compared to controls (adjusted p-value = 0.0339).

These findings highlight the complexity of epigenetic changes in AD, as well as the need for further research to clarify the relationship between epigenetic age acceleration and cognitive dysfunction. The observed deceleration of epigenetic aging in AD patients may be explained in part by the older age of the cohort [71], but future research is necessary to support this hypothesis.

### Limitations of array-based clocks; batch effects

The choice of analytical platform is critical for epigenetic studies, as highlighted by recent comparative analyses between the Illumina Infinium HumanMethylation450K and MethylationEPIC BeadChip arrays. These studies have shown substantial variability in methylation measurements, particularly at sites with low methylation variance, underscoring a fundamental limitation of array technologies that can be exacerbated by batch effects and dependency on pre-designed probes (Cheung, Burgers, Young, Cockell, & Reynard, 2020).

Batch effects are a significant concern in genomic technologies, especially in epigenome-wide association studies using Illumina Infinium Methylation BeadChips, where they can diminish experimental power and lead to false positives. Despite employing batch-effect removal tools like ComBat and Harman on various datasets, residual batch effects persist, particularly related to the processing day, glass slide, and array position, affecting thousands of probes and complicating the interpretation of epigenetic studies (Ross et al., 2022). Additionally, there is a growing recommendation in the field of epigenetic age calculation using array technologies that data should not be normalized nor corrected for batch effects. This advice stems from the need to preserve the integrity of longitudinal studies where epigenetic age is monitored over time, a major application of this metric beyond single-timepoint clinical studies. The capability to consistently measure epigenetic age across multiple time points is crucial for assessing the effects of lifestyle changes and other interventions. However, the persistent batch effects and the necessity for normalization challenge the utility of array-based platforms for such longitudinal monitoring, underscoring the advantages of using targeted NGS for ongoing epigenetic age analysis.

In contrast, targeted next-generation sequencing (NGS) provides a more robust alternative. It offers higher resolution by sequencing individual DNA molecules, which allows for the detection of methylation profiles of the DNA molecules rather than just averages. NGS platforms are typically less prone to batch effects due in part to the sequencing process itself, which does not rely on the hybridization conditions that can vary in array-based platforms.

However, since next-generation sequencing captures profiles of each DNA molecule independently and takes into consideration the heterogeneity of DNA methylation profiles of single DNA molecules even in the same tissue, deep reads are required to have sufficient statistical power to accurately represent the diversity of DNA methylation profiles. Clinical tests need to be cost-effective and robust. Targeted amplification and Next-generation sequencing of thousands or even hundreds of regions with high depth and accuracy is highly technical, demanding and extremely costly and makes it unfeasible as a widespread robust clinical test. It is, therefore, imperative that a clinically widely used next-generation sequencing-based test be parsimonious and nimble and utilize the lowest number of regions amplified and sequenced. We, therefore, performed in this study a thorough analysis to determine whether it is possible to develop a clock that is based on the smallest number of DNA regions without compromising its correlation with age and its sensitivity to clinical states that affect aging. Our analysis revealed that the *ELOVL2* region on its own performs as well or is even superior to other clocks that use hundreds or even thousands of sites. We then developed a robust next-generation sequencing assay of a region that contains the CpGs included in the EpiAgePublic model as well as the other 10 CpGs.

We observed remarkably low coefficients of variation (CVs) across the first nine CpG sites, with CVs ranging from 0.28% to 6.7%, demonstrating NGS’s robustness in accurate methylation profiling. Even for CpG sites with higher CVs, which ranged from 1% to 25% at sites of lower methylation levels, NGS shows a more consistent performance compared to arrays. Illustrated in Figure 6A, these results highlight that NGS not only offers higher resolution and greater specificity but also exhibits reduced susceptibility to batch effects. This independence from hybridization conditions and pre-designed probes allows for a more comprehensive and flexible analysis, making NGS particularly valuable for precise and reproducible methylation profiling across diverse biological samples and time points. This technological superiority enhances our ability to monitor epigenetic changes with greater accuracy and reliability, which is crucial for longitudinal studies and the assessment of lifestyle or other interventions over time.

### Advantages and limitations of simplifying epigenetic clocks with EpiAgePublic

While traditional epigenetic clocks rely on analyzing hundreds to thousands of CpG sites, EpiAgePublic achieves comparable predictive accuracy with significantly fewer sites. This reduction in complexity could lead to fewer errors associated with probe variability and hybridization inefficiencies inherent in array platforms. However, it is essential to acknowledge that the broader genomic coverage of traditional clocks may capture a more comprehensive epigenetic signature of aging. Therefore, while EpiAgePublic offers an efficient alternative, it should be seen as complementary to existing methods rather than a replacement.

The primary limitation of this study is the potential oversimplification of the aging process using only three CpG sites. Aging is a multifactorial process influenced by numerous genetic, environmental, and lifestyle factors. Therefore, further studies are needed to validate the effectiveness of EpiAgePublic across larger and more varied populations. Additionally, longitudinal studies would help in understanding how EpiAgePublic responds to changes over time and under different physiological or pathological conditions.

Moreover, the integration of EpiAgePublic with other biological markers of aging, such as telomere length, oxidative stress markers, and inflammatory cytokines, could provide a more holistic view of the aging process. This integrative approach could lead to the development of a multi-dimensional aging model that combines genetic, epigenetic, and biochemical indicators of age.

## Supplementary Material

Supplementary Tables
