# Correction for: EpiAge: a next-generation sequencing-based *ELOVL2* epigenetic clock for biological age assessment in saliva and blood across health and disease

**DOI:** 10.18632/aging.206263

**Published:** 2025-05-31

**Authors:** David Cheishvili, Sonia Do Carmo, Filippo Caraci, Margherita Grasso, A Claudio Cuello, Moshe Szyf

**Affiliations:** 1 EpiMedTech Global, Singapore 409051, Singapore; 2 HKG Epitherapeutics Ltd., Hong Kong SAR, China; 3 Gerald Bronfman Department of Oncology, McGill University, Montreal H4A 3T2, Canada; 4 Department of Pharmacology & Therapeutics, McGill University, Montreal H3G 1Y6, Canada; 5 Visiting Professor, Department of Pharmacology, Oxford University, Oxford OX13QT, UK; 6 Department of Drug and Health Sciences, University of Catania, Catania 95125, Italy; 7 Neuropharmacology and Translational Neurosciences Research Unit, Oasi Research Institute-IRCCS, Troina 94018, Italy

**Keywords:** epigenetic clock, elovl2, next-generation sequencing, EpiAge, Alzheimer’s disease

**This article has been corrected:** The authors recently found that the formula for the EpiAgePublic model shown in the “Development and application of the EpiAgePublic model to investigate biological aging dynamics” section contains a typographical error. Specifically, the coefficient for cg24724428 was incorrectly presented as **–30.44**, whereas the correct value is **+30.44**.

The model calculates beta values using the formula:


$$\left(\beta_{cg\text{16867657}}\times \text{122}\text{.70}+\beta_{cg\text{21572722}}\times \text{24}\text{.45}+\beta_{cg\text{24724428}}\times \text{30}\text{.44}\right)-\text{42}\text{.91}\text{.}$$

The authors stated that this correction does not affect the experimental outcome or conclusions of the study. The authors sincerely apologize for any inconvenience caused.

