# Supplementary material for: EpiAge: a next-generation sequencing-based ELOVL2 epigenetic clock for biological age assessment in saliva and blood across health and disease
Source: Aging (Albany NY). 2025 Jan 22;17(1):131–60. doi: 10.18632/aging.206188 (PMC11810066; doi:10.18632/aging.206188)
Supplement: Supplementary Tables [file aging-17-206188-s001.pdf]

SUPPLEMENTARY TABLES

Supplementary Table 1. Regression analysis results for chronological age across various epigenetic clocks and CpG sites including confounder adjustments.

Model description	R^2	Adj. R^2	F-Stat	p(F-stat)	Age Coef	Std Err	t-Value	p-Value	95% CI Lower	95% CI Upper	AIC	BIC	Observations
cg16867657 (*ELOVL2* site)	0.757	0.757	1801	<0.0001	0.0047	4.50E-05	105.465	<0.0001	0.005	0.005	-17790	-17730	4625
cg21572722 (*ELOVL2* site)	0.702	0.702	1362	<0.0001	0.0025	3.04E-05	82.951	<0.0001	0.002	0.003	-21430	-21370	4625
cg24724428 (*ELOVL2* site)	0.626	0.625	965.1	<0.0001	0.0039	4.87E-05	79.2	<0.0001	0.004	0.004	-17060	-17010	4625
EpiAgePublicBlood	0.779	0.778	2030	<0.0001	0.7612	0.007	111.887	<0.0001	0.748	0.775	28630	28690	4625
DNAmAge (Horvath)	0.842	0.841	3067	<0.0001	0.78	0.005	145.819	<0.0001	0.77	0.791	26410	26470	4625
DNAmAge Hannum	0.883	0.883	4357	<0.0001	0.8554	0.006	153.678	<0.0001	0.845	0.866	26780	26830	4625
DNAmPhenoAge	0.787	0.786	2127	<0.0001	0.8966	0.008	116.929	<0.0001	0.882	0.912	29740	29800	4625
DNAmAgeSkinBloodClock	0.898	0.898	5078	<0.0001	0.8855	0.005	184.542	<0.0001	0.876	0.895	25400	25460	4625
DNA GrimAge v1	0.806	0.806	2395	<0.0001	0.689	0.006	121.39	<0.0001	0.678	0.7	26960	27010	4625
DNA GrimAge v2	0.765	0.765	1882	<0.0001	0.6501	0.006	108.32	<0.0001	0.638	0.662	27470	27530	4625

Supplementary Table 2. Summary table for replicates.

ID	Mean	SD	CV (%)	95% CI	Minimum	Maximum	Median	25th Percentile	75th Percentile
1	67.3	1.34	1.99%	[65.17, 69.43]	66.09	68.72	67.2	66.13	68.58
2	90.67	0.99	1.09%	[89.10, 92.24]	89.38	91.77	90.77	89.7	91.55
3	63.47	1.56	2.46%	[60.99, 65.95]	61.65	65.33	63.44	61.97	64.99
4	80.19	1.04	1.29%	[78.54, 81.84]	79.51	81.72	79.76	79.54	81.26
5	72.89	1.6	2.19%	[70.35, 75.43]	71.5	74.44	72.8	71.5	74.35
6	72.61	4.43	6.10%	[65.56, 79.67]	66.57	76.47	73.71	67.96	76.18
7	83.37	1.25	1.50%	[81.38, 85.36]	82.04	84.84	83.3	82.2	84.61
8	86.78	2.04	2.35%	[83.54, 90.03]	84.93	89.21	86.49	85.02	88.83
9	66.6	0.98	1.48%	[65.03, 68.16]	65.58	67.73	66.54	65.68	67.57
10	74.35	2.6	3.49%	[70.22, 78.48]	71.28	77.41	74.36	71.84	76.85
11	78	1.01	1.29%	[76.40, 79.60]	76.91	79.11	78	77.04	78.97
12	79.25	1.65	2.08%	[76.62, 81.87]	77.56	81.29	79.07	77.75	80.92
13	87.51	0.85	0.98%	[86.15, 88.87]	86.59	88.64	87.41	86.76	88.37
14	62.81	1.24	1.98%	[60.83, 64.79]	61.1	63.94	63.1	61.51	63.82
15	81.45	1.52	1.86%	[79.03, 83.86]	79.65	83.28	81.43	80	82.91
16	82.22	1.88	2.29%	[79.23, 85.21]	79.59	84.02	82.62	80.29	83.74
17	82.62	1.47	1.78%	[80.28, 84.96]	81.62	84.81	82.03	81.72	84.11
18	70.94	1.8	2.54%	[68.07, 73.81]	69.39	73.07	70.64	69.42	72.75
19	77.23	1.14	1.48%	[75.41, 79.05]	75.67	78.25	77.5	76.03	78.16
20	87.44	2.78	3.18%	[83.02, 91.86]	84.12	90.63	87.5	84.72	90.09
21	72.08	0.79	1.09%	[70.83, 73.33]	71.4	73.21	71.85	71.51	72.87
22	77.4	0.67	0.87%	[76.33, 78.47]	76.86	78.35	77.19	76.9	78.11
23	88.47	2.45	2.77%	[84.57, 92.37]	86.01	91.31	88.28	86.23	90.9
24	66.71	1.58	2.37%	[64.19, 69.22]	65.26	68.7	66.44	65.36	68.34
25	70.23	2.79	3.98%	[65.78, 74.67]	67.92	73.78	69.61	67.96	73.12
26	68.85	1.19	1.73%	[66.95, 70.74]	67.78	70.23	68.69	67.82	70.03
27	78.66	2.4	3.05%	[74.85, 82.48]	75.61	81.41	78.81	76.31	80.86
28	80.28	0.76	0.94%	[79.08, 81.49]	79.59	81.14	80.2	79.62	81.03

29	64.02	1.97	3.08%	[60.88, 67.15]	62.06	66.69	63.66	62.35	66.05
30	76.23	1.19	1.56%	[74.35, 78.12]	75.15	77.8	75.99	75.24	77.47
31	65.66	1.76	2.68%	[62.86, 68.46]	64.11	67.2	65.66	64.12	67.19
32	70.15	1.95	2.78%	[67.05, 73.25]	68.06	72.41	70.07	68.32	72.07
33	69.16	0.82	1.19%	[67.85, 70.47]	67.97	69.79	69.44	68.29	69.75
34	66.46	2.03	3.05%	[63.24, 69.69]	64.71	69.29	65.93	64.87	68.59
35	68.06	0.72	1.06%	[66.92, 69.20]	67.25	68.72	68.13	67.36	68.69
36	81.62	1.52	1.86%	[79.20, 84.03]	80.2	83.75	81.26	80.42	83.18
37	74.34	1.66	2.24%	[71.69, 76.98]	72.53	75.93	74.44	72.73	75.84
38	66.67	1.71	2.57%	[63.95, 69.39]	64.57	68.56	66.77	64.96	68.27
39	72.9	0.42	0.57%	[72.24, 73.56]	72.47	73.41	72.85	72.52	73.32
40	68.86	2.32	3.37%	[65.16, 72.55]	66.68	72.11	68.32	67	71.26
41	67.18	1.25	1.86%	[65.19, 69.17]	65.78	68.63	67.15	65.98	68.4
